# CCL11/CCR3-dependent eosinophilia alleviates malignant pleural effusions and improves prognosis

**DOI:** 10.1038/s41698-024-00608-8

**Published:** 2024-06-29

**Authors:** Min Zhang, Lixia Xia, Wenbei Peng, Guogang Xie, Fei Li, Chao Zhang, Madiha Zahra Syeda, Yue Hu, Fen Lan, Fugui Yan, Zhangchu Jin, Xufei Du, Yinling Han, Baihui Lv, Yuejue Wang, Miao Li, Xia Fei, Yun Zhao, Kaijun Chen, Yan Chen, Wen Li, Zhihua Chen, Qiong Zhou, Min Zhang, Songmin Ying, Huahao Shen

**Affiliations:** 1 Department of Respiratory and Critical Care Medicine, Key Laboratory of Respiratory Disease of Zhejiang Province, The Second Affiliated Hospital of Zhejiang University School of Medicine, Hangzhou, 310009 Zhejiang China; 2 Department of Respiratory and Critical Care Medicine, Union Hospital, Tongji Medical College, Huazhong University of Science and Technology, Wuhan, 430022 China; 3 Department of Respiratory and Critical Care Medicine, Shanghai General Hospital, Shanghai Jiao Tong University School of Medicine, Shanghai, 200080 China; 4 International Institutes of Medicine, The Fourth Affiliated Hospital of Zhejiang University School of Medicine, Yiwu, 322000 China; 5 Department of Pharmacology, Zhejiang University School of Medicine, Hangzhou, 310058 China; 6 State Key Lab for Respiratory Diseases, National Clinical Research Centre for Respiratory Disease, Guangzhou, 510120 Guangdong China

**Keywords:** Cancer microenvironment, Cancer immunotherapy

## Abstract

Malignant pleural effusion (MPE) is a common occurrence in advanced cancer and is often linked with a poor prognosis. Eosinophils were reported to involve in the development of MPE. However, the role of eosinophils in MPE remains unclear. To investigate this, we conducted studies using both human samples and mouse models. Increased eosinophil counts were observed in patients with MPE, indicating that the higher the number of eosinophils is, the lower the LENT score is. In our animal models, eosinophils were found to migrate to pleural cavity actively upon exposure to tumor cells. Intriguingly, we discovered that a deficiency in eosinophils exacerbated MPE, possibly due to their anti-tumor effects generated by modifying the microenvironment of MPE. Furthermore, our experiments explored the role of the C-C motif chemokine ligand 11 (CCL11) and its receptor C-C motif chemokine receptor 3 (CCR3) in MPE pathology. As a conclusion, our study underscores the protective potential of eosinophils against the development of MPE, and that an increase in eosinophils through adoptive transfer of eosinophils or increasing their numbers improved MPE.

## Introduction

Malignant pleural effusion (MPE) can be caused by the primary malignant pleural mesothelioma or metastatic tumors from the lung, breast or elsewhere^1^. It is characterized by large accumulations of fluid, which may include tumor tissue or cells, and has an average survival of 3–12 months^2,3^. Chemotherapy may be used as the first line of treatment for MPE with the goals of tumor reduction and pleural fluid absorption^4^. However, many MPE patients are not eligible for chemotherapy because of their causative tumors or chemoresistance^4^. Current therapies are palliative and often ineffective, and have not led to significant improvements in terms of survival^5–7^.

Clinical studies show MPE patients have a highly immunosuppressive tumor microenvironment (TME) with numerous immunosuppressive cells and mediators^8–11^. TME factors can induce immune cell exhaustion, and reprogramming or removing them may improve immunotherapy^12^. MPE’s immune landscape is under investigation from clinical and prognostic perspectives. Intrapleural injections of anti-PD1 monoclonal antibodies may control MPE and tumor growth^13^. Intratumorally administered CAR-T cells, superior to intravenous ones, deeply penetrate pleural tumors and limit infiltration and activation. Rapid expansion and function of CAR-T cells then overcome suppressive TME, causing tumor regression and eradication at lower doses^14^. Targeting the local immune system could be promising for the MPE management.

Eosinophils are evolutionarily conserved and pleiotropic cells that display key effector functions in allergic diseases, such as asthma^15–17^. Recent mechanistic studies utilizing novel experimental methods have upgraded this conventional definition. They also play crucial roles in the fields of metabolism, tissue regeneration, immunity, and cancer^18–20^. Current research indicates that, depending on their niche, eosinophils may have either a pro- or anti-tumor effect in cancer^20^. In pulmonary metastases of breast cancer, lymphocyte-mediated antitumor immunity may be enhanced by recruited eosinophils^21^. In immunodeficient NPG mice with HepG2 and HCT116 cell injection, eosinophils derived from human pluripotent stem cells (hPSCs), together with CAR-T cells, revealed potential synergistic effects in decreasing tumor growth and boosting mouse survival^22^. In syngeneic and genetic models of colorectal cancer (CRC), the GM-CSF–IRF5 signaling axis in eosinophils promotes antitumor immunity through activation of type 1 T cell responses^23^. In another colorectal cancer model, tumor-infiltrating eosinophils have an antitumorigenic activity in vivo and their functions can be distinguished from cytotoxic T cells and intratumoral macrophages^24^. We recently discovered that eosinophils promoted CCL6-dependent metastatic tumor growth in the context of inflammation^25^. However, our knowledge of eosinophils’ role in MPE is still developing. There have been very few published investigations, and the findings are conflicting^26,27^. In immunocompetent, interleukin 5-deficient (*Il5*^–/–^) mice with intrapleurally injected tumor cells, MPE formation significantly decreased. Reduced eosinophils and myeloid-derived suppressor cells (MDSCs) levels were linked to MPE mitigation^26^. Conversely, a retrospective study showed lung cancer patients with eosinophilic pleural effusion (EPE) had better prognosis than non-EPE patients^27^. However, it remains unclear how eosinophils are recruited into pleural cavity and what role they play in diverse types of MPE.

In this investigation, we describe a coincidental finding that eosinophils increased in pleural cavity after injection of LLC, MC38, and 4T1 tumor cells. In a similar manner, elevated eosinophil levels observed in MPE patients. Through the utilization of Eos-null mice, we have uncovered that eosinophils exhibit a protective function in MPE by modifying its microenvironment. Our findings further show that CCL11 and CCR3 contributed to eosinophil recruitment in pleural cavity and limit MPE formation. Additionally, adoptive transfer of eosinophils or increasing eosinophil numbers may suggest an effective approach for the disease.

## Results

### Eosinophils in patients with MPE

**Fig. 1:**
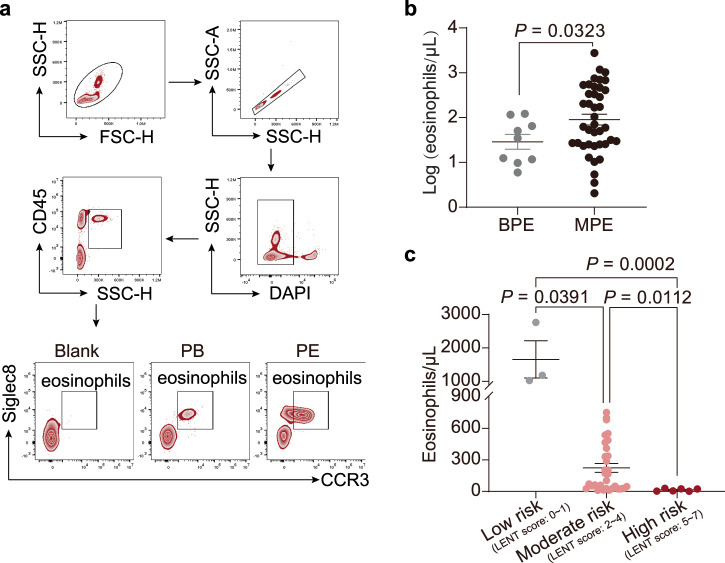
Eosinophils increased in patients with MPE. **a** Flow cytometry hierarchical gating strategy for eosinophils in human. **b** Summary of Log (eosinophils/μL) in BPE (*n* = 10) and MPE (*n* = 40). **c** The number of eosinophils in the low risk group, moderate risk group, and high risk group (according to the LENT score) was determined in MPE. Data are shown as mean ± SEM and were analyzed using unpaired Student’s *t* test for (**b**) and one-way ANOVA followed by Bonferroni test for (**c**). *P* values. *P* < 0.05 was considered statistically significant differences. Abbreviations: MPE, malignant pleural effusion; BPE, benign pleural effusion; FC, flow cytometry; LENT, Lactate dehydrogenase, Eastern Cooperative Oncology Group (ECOG) performance status (PS), Neutrophil-to-lymphocyte ratio (NLR), Tumor type.

To examine eosinophil distribution in MPE patients, our study included 40 MPE patients and 10 BPE patients from three hospitals, with a mean age of 69 ± 8 years (MPE) and 59 ± 8 years (BPE) (Supplementary Table 1). Using flow cytometry, we quantified eosinophils marked as DAPI^-^CD45^+^SSC^hi^CCR3^+^Siglec8^+^ (Fig. 1a) in MPE and BPE. We observed higher eosinophil frequencies in MPE than BPE (Fig. 1b). MPE patients with lower eosinophils had higher LENT (Lactate Dehydrogenase, Eastern Cooperative Oncology Group performance status, Neutrophil-to-lymphocyte ratio, Tumor type) score (Fig. 1c), which calculates prognosis^28^. Thus, eosinophils infiltrate into MPE and negatively correlate with the LENT score.

### Eosinophils distribution during the formation of MPE in mice

**Fig. 2:**
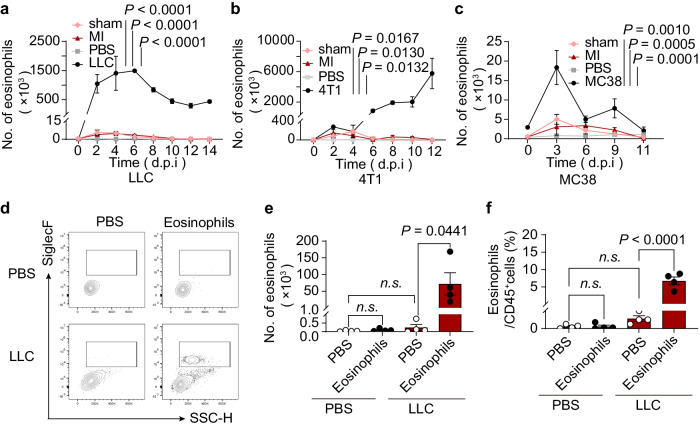
Accumulation of eosinophils during MPE Formation in Mice. **a-c** FC analysis was used to determine the eosinophil counts in pleural lavage fluid or pleural effusions in WT mice over time during the development of LLC-MPE (**a**), 4T1-MPE (**b**), and MC38-MPE **(c**), *n* = 2–8 per group. **d**–**f** Eosinophils were actively recruited in pleural cavity. Highly purified eosinophils from the PB of *Il5* Tg mice were adoptively transferred into Eos-null mice with or without the LLC-MPE model, and the eosinophils counts in pleural lavage fluid were assessed at day 3. **d** Representative flow cytometry plot of eosinophils. The number of eosinophils (**e**) and the percentage of eosinophils/CD45^+^ cells (**f**) in Eos-null mice. *n* = 4 per group. Data are presented as the mean ± SEM. Two-way ANOVA followed by Bonferroni test for (**a**, **b**, **c**, **e**, **f**). *P* < 0.05 indicated statistically significant differences. Abbreviations: sham, sham-operated; MI myocardial infarction; *n.s*., not significant.

Next, we used LLC tumor cells, 4T1 breast tumor cells, or MC38 colon adenocarcinoma cells to induce MPE in WT mice. Eosinophil infiltration in pleural cavity of LLC-MPE bearing mice was shown by immunofluorescent labeling with anti-eosinophil peroxidase (EPX) antibody, as compared to PBS-treated mice (Supplementary Fig. 1a). Flow cytometry investigation of eosinophils in pleural cavity of tumor cell-induced MPE mice identified them as DAPI^-^CD45^+^Gr1^-/low^CD11c^-^CD11b^+^F4/80^+^SiglecF^+^SSC^hi^ cells and were clearly distinguishable from all other leukocyte populations (Supplementary Fig. 1b). Pleural cavity’s eosinophil count fluctuated over time (Supplementary Fig. 1c). The eosinophils recruitment emerged as an early event during the progression of LLC-MPE (Fig. 2a). Following inoculation, eosinophils were among the prominent leukocyte populations alongside macrophages/monocytes, lymphocytes, and neutrophils (Supplementary Fig. 1d). However, as the pleural tumors grew and pleural fluid formed at later stages, the counts of eosinophils declined, thereby allowing macrophages to assume a more dominant presence (Supplementary Fig. 1d). MC38-MPE (Fig. 2c) also experienced this behavior. However, the eosinophils recruitment was a slightly late event in the course of 4T1-MPE (Fig. 2b). The control group for the aforementioned experiment used pleural lavage fluid from mice with MI (Supplementary Fig. 1e). Overall, these findings imply that different kind of tumor cells influence the eosinophil infiltration and distribution in pleural cavity during the formation of MPE in mice.

To investigate eosinophil accumulation mechanisms in pleural cavity after tumor cell injection, we conducted a chemotaxis assay using LLC cell-conditioned media or pleural lavage fluid in vitro. Results indicated enhanced eosinophil migratory capacity upon stimulation (Supplementary Fig. 2a, b). We also adoptively transferred eosinophils from *Il5* Tg mice to PBS-treated or LLC-bearing Eos-null mice and observed significant eosinophil recruitment in pleural cavity post-LLC injection in vivo (Fig. 2d, e) and similar results in CD45.1^+^ mice (Supplementary Fig. 2c-e). These findings suggest that eosinophils are actively recruited in pleural cavity.

### Eosinophil depletion promoted the progression of MPE

**Fig. 3:**
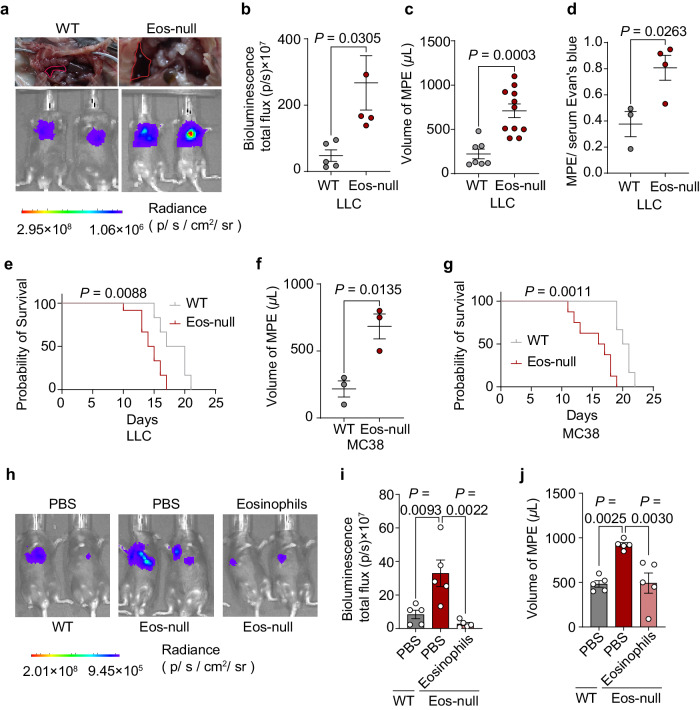
Impact of eosinophil deficiency on MPE. **a**–**g** WT and Eos-null mice were injected intrapleurally with LLC or MC38 cells, and MPE was measured at day 14. **a** Representative images of effusions and bioluminescence analysis after LLC-Luc injection. **b** Bioluminescence at day 14 after LLC-Luc injection. *n* = 5 per group. **c** Volume of effusion in LLC-MPE model. *n* = 8–13 per group. **d** Pleural permeability in LLC-MPE model. *n* = 3 per group. **e** Survival curve of WT and Eos-null mice in LLC-MPE model. *n* = 7–15 per group. **f** Volume of MPE in WT and Eos-null mice after MC38 cell injection. *n* = 3 per group. **g** Survival curve of WT and Eos-null mice in MC38-MPE model. *n* = 7–15 per group. **h**–**j** Eos-null mice received eosinophil injections after LLC-Luc injection and MPE was measured at day 14. **h** Representative bioluminescence images. **i** Bioluminescence at day 14. *n* = 5 per group. **j** Volume of effusion. *n* = 5 per group. Data are mean ± SEM. *P* < 0.05 were considered significant. Data are shown as the mean ± SEM. Unpaired Student’s *t* test for (**b**, **c**, **d**, **f**); one-way ANOVA followed by Bonferroni test for (**i**, **j**). Kaplan–Meier survival statistical analysis was performed using the log-rank test.

To examine the role of eosinophils, we used Eos-null mice and their littermate control mice with MPE. Eos-null mice lack eosinophils and transgenically express diphtheria toxin under the eosinophil peroxidase promoter^29^. Interestingly, eosinophil deficiency in the LLC model aggravated MPE, including pleural tumor burden (Fig. 3a, b), MPE volume (Fig. 3a, c), pleural permeability (Fig. 3d), and the survival (Fig. 3e). Similar results were observed in the MC38 model (Fig. 3f, g). To further substantiate the protective function of eosinophils, we adoptively transferred eosinophils to the Eos-null mice and the data revealed a reduction in the volume of MPE and the pleural tumor burden (Fig. 3h–j). These findings show that eosinophils could inhibit the MPE.

To explore the eosinophils anti-tumor activities, we investigated their potential for direct anti-tumor activities towards tumor cells and their ability to orchestrate the participation of other immune cells in pleural cavity. With different co-culture ratios, neither non-contact nor contact co-culture of eosinophils with LLC, 4T1 or MC38 cells resulted in tumor cell death (Supplementary Fig. 3a–d). No differences in the expression of apoptosis marker cleaved-caspase 3 and proliferation marker Ki67 were observed in the pleural tumor between Eos-null and WT mice (Supplementary Fig. 3e–g). Following that, we examined the immune cell profile of pleural cavity seven days after LLC injection in WT and Eos-null mice (Supplementary Table 2), and the markers expressed in each sample were shown in the Supplementary Table 3. Because of the stimulation by PMA/BFA/Ion, the proportion of each cluster is not exact. We discovered that the Eos-null group had a lower percentage of NK cells, macrophages/monocytes, DC cell clusters, and was devoid of eosinophils when compared to the WT group (Supplementary Fig. 4a, b). The surprising discovery prompted additional analysis of the CD3^+^ T cells, which revealed that the Eos-null group had reduced Th17 and Th1 cells and higher proportions of Treg cells (Supplementary Fig. 4c, d). Remarkably, we were unable to find Th2 cells in this situation. In the Eos-null group, CD3^+^ T cells expressed less proliferation marker Ki67 and activation marker CD69 (Supplementary Fig. 4e). In summary, these data show that pleural cavity-infiltrating eosinophils do not act directly on tumor cells, but rather probably orchestrate additional immune cells such as NK cells, DC cells, macrophages/monocytes, and T cells to participate in the anti-tumor response.

### CCR3 played a crucial role in eosinophil recruitment

To characterize pleural cavity eosinophils, we conducted RNA-sequencing and compared it to naïve bone marrow eosinophils (Supplementary Fig. 5a–c), identifying 5807 differentially expressed genes (*P* < 0.05, |log2 fold change | >1). Among them, 1127 were upregulated and 4680 downregulated in pleural cavity eosinophils compared to naïve bone marrow eosinophils (Supplementary Fig. 5d). Gene set enrichment analysis (GSEA) of differentially expressed genes indicated involvement in C-C motif chemokine receptor binding, eosinophil chemotaxis, cell killing regulation, chemokine responsiveness, and IFN-α/β signaling (Supplementary Fig. 5e). Upstream regulator analysis (URA) using ingenuity pathway analysis (IPA) identified central upstream regulators like IFN-γ, TNF-α, IL-21, and IFN-α/β (Supplementary Fig. 5f). Moreover, IRF7, IRF3, and GATA1 were top predicted upstream transcription regulators (Supplementary Fig. 5g) (normalized *P* < 0.05, |NES | >1).

**Fig. 4:**
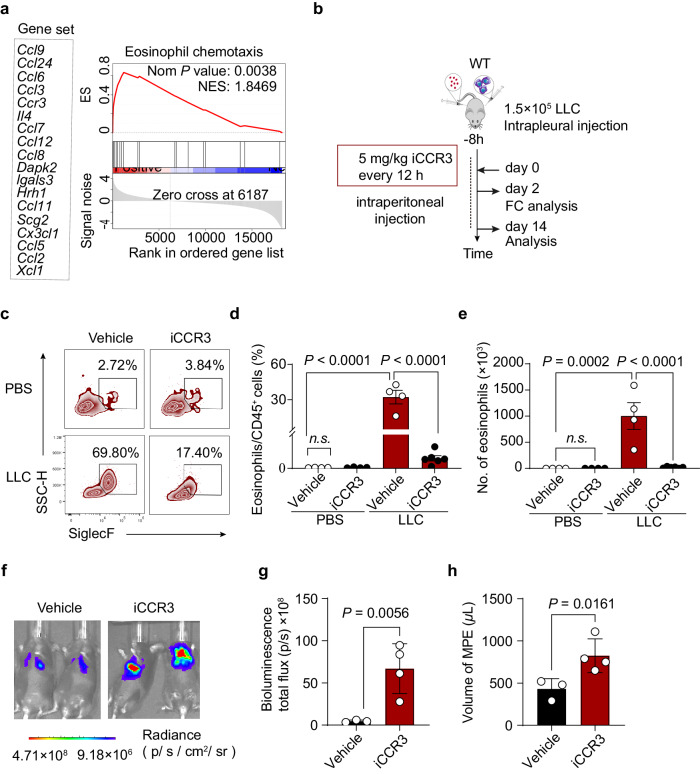
Eosinophils recruitment facilitated by CCR3. **a** Eosinophils from bone marrow and pleural lavage fluid at day 2 were sorted by MACS and FACS and subjected to RNA sequencing to profile gene expression. The enriched eosinophil chemotaxis pathway gene set is shown (NES = 1.846; NOM *P* = 0.003). **b**–**h** Blockade of CCR3 inhibited eosinophil recruitment, promoted pleural tumor growth, and increased MPE volume. **b** Experimental design of iCCR3. **c** Representative flow cytometry diagram of eosinophils in pleural lavage fluid. Eosinophils were identified as DAPI^-^CD45^+^Gr1^-/low^CD11c^-^CD11b^+^F4/80^+^SiglecF^+^SSC^hi^ cells. **d**, **e** Number of eosinophils and percentage of eosinophils/CD45^+^ cells. **f** Representative images of bioluminescence analysis. **g**, **h** Bioluminescence measured as photons per second (p/s) and effusion volume recorded. Data are mean ± SEM. Two-way ANOVA followed by Bonferroni test for (**d**, **e**) two-tailed unpaired Student’s *t* test for (**g**, **h**). *P*< 0.05 were considered significant (*n* = 4 per group). Abbreviations: *n.s*. not significant.

To understand the mechanism of eosinophil recruitment into pleural cavity, we analyzed gene sets correlated with eosinophil chemotaxis, identifying 18 enriched genes (Fig. 4a). We confirmed that >95% of CCR3^+^ cells in pleural cavity were eosinophils (Supplementary Fig. 6). Using the SB297006 to knockdown CCR3 in MPE models (Fig. 4b), we observed impaired eosinophil recruitment (Fig. 4c–e), increased pleural tumor burden (Fig. 4f, g), and MPE volume (Fig. 4h). These findings suggest that CCR3 plays a crucial role in eosinophil recruitment into pleural cavity, inhibiting MPE formation.

### Strategies for increasing eosinophils

**Fig. 5:**
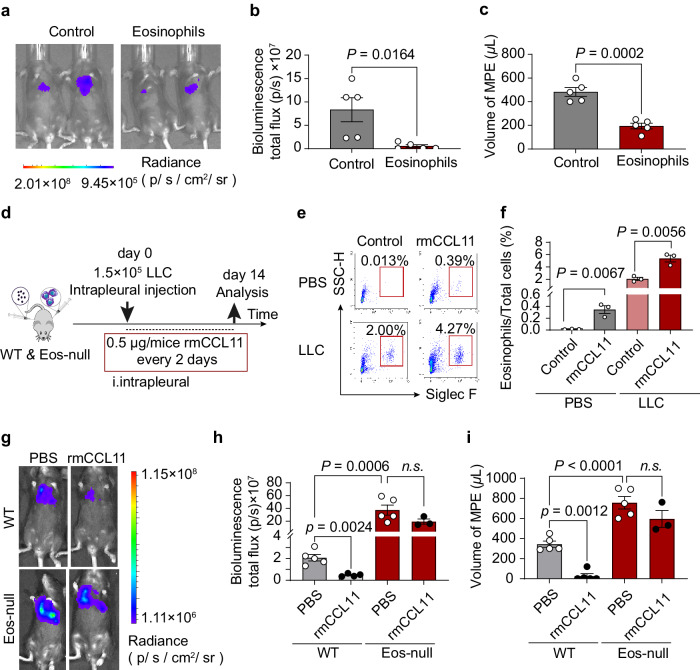
MPE improved by increasing eosinophils via adoptive transfer and intrapleural injection of rmCCL11. **a**–**c** WT mice received eosinophils through adoptive transfer. **a** Representative images of bioluminescence analysis. **b**, **c** Quantitative photon counts within the chest area and the volume of pleural effusions. *n* = 5 per group. **d**–**i** Intrapleural injection of rmCCL11 promoted eosinophil recruitment, inhibited pleural tumor growth, and decreased MPE volume. **d** Experimental design schematic. **e** Representative flow cytometry diagram of eosinophils in pleural lavage fluid. Eosinophils were identified as DAPI^-^CD45^+^Gr1^-/low^CD11c^-^CD11b^+^F4/80^+^SiglecF^+^SSC^hi^ cells. **f** Number of eosinophils and percentage of eosinophils/total cells. *n* = 3 per group. **g** Representative images of bioluminescence analysis. **h**, **i** Quantitative photon counts within the chest area and the volume of pleural effusion. Data are mean ± SEM. Two-tailed unpaired Student’s *t* test for (**b**, **c**) two-way ANOVA followed by Bonferroni test for (**f**, **h**, **i**). *P* < 0.05 were considered significant. Abbreviations: *n.s*. not significant.

To explore eosinophils as a potential MPE therapy, we employed several methods. First, transferring eosinophils to WT mice reduced MPE volume and pleural tumor burden (Fig. 5a–c). We then measured 200 cytokines and found CCL11 (eotaxin1), a CCR3 ligand, significantly increased in Eos-null mice compared to WT controls (Supplementary Fig. 7a). Other CCR3 ligands showed no differences (Supplementary Table 4). ELISA results revealed higher CCL11 levels in LLC-MPE mice than the control and PB (Supplementary Fig. 7b). CCL11 was also elevated in MPE patients compared to BPE (Supplementary Fig. 7c) and positively correlated with eosinophils counts in MPE patients (Supplementary Fig. 7d).

In view of the above findings, another strategy of intrapleural injection with recombinant mouse CCL11 (rmCCL11) was conducted to increase the number of eosinophils (Fig. 5d). Intrapleural injection of rmCCL11 (0.5 μg/mice) increased eosinophils recruitment in pleural cavity following the LLC tumor cells challenge (Fig. 5e, f). In addition, pleural tumor burden and MPE volume were reduced in LLC-MPE mice after intrapleural injection of rmCCL11 (0.5 μg/mice) (Fig. 5g–i).

**Fig. 6:**
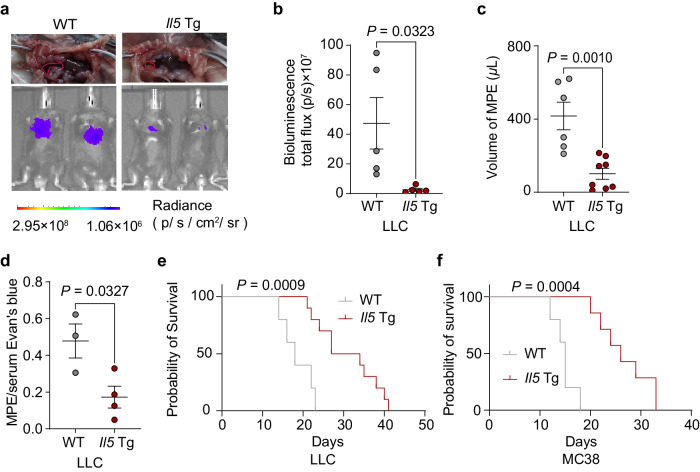
Increased eosinophil production by genetic engineering of mice for MPE treatment. **a** Representative images of effusions and bioluminescence analysis at day 14 after pleural tumor challenge with LLC-Luc. **b** Bioluminescence measured as photons per second (p/s). *n* = 5 per group. **c** Volume of effusion. *n* = 5–8 per group. **d** Pleural permeability assay performed by calculating the MPE/serum Evan’s blue ratio in the LLC-MPE model. *n* = 3–4 per group. **e**–**f** Survival curves of WT mice and *Il5* Tg mice in the LLC-MPE (**e**) and MC38-MPE (**f**) models. *n* = 7–15 per group. Data are shown as mean ± SEM. Two-tailed unpaired Student’s *t* test for (**b**–**d**). Kaplan–Meier survival statistical analysis was performed using the log-rank test (**e**–**f**). *P* < 0.05 were considered statistically significant.

Using *Il5* Tg mice, which constitutively overexpress *Il5* in T cells^30^, we achieved continuous eosinophil production. Eosinophilia reduced pleural tumor burden, MPE volume, and permeability in LLC-MPE mice (Fig. 6a–d), doubling survival duration (Fig. 6e, f). No difference in cleaved-caspase 3 and Ki67 was observed between groups (Supplementary Fig. 8a–c).

Overall, our results suggest that increasing eosinophils through adoptive transfer of eosinophils or increasing eosinophil numbers may be a promising therapeutic approach for mitigating MPE.

## Discussion

The precise role of eosinophils in MPE remains ambiguous. Our investigation revealed an accumulation of eosinophils in MPE patients, where higher eosinophil counts corresponded to lower LENT scores. Additionally, we observed a dynamic recruitment of eosinophils to pleural cavity in MPE mice. Notably, the absence of eosinophils aggravated MPE by altering the microenvironment, while the exacerbation of the condition occurred with the use of CCR3 inhibitors, which prevent eosinophil entry. In addition, interventions involving adoptive transfer or increased eosinophil numbers showed a reduction in MPE partly.

Previous research has consistently shown higher eosinophil levels in both MPE patients and MPE mice^25,26^. Across various models (LLC-MPE, MC38-MPE, and 4T1-MPE), we observed distinct variations in eosinophil dynamics within pleural cavity. That might be attributed to the unique biological characteristics exhibited by different tumor cell types. Interestingly, research on eosinophils’ role in MPE presents conflicting findings^26,27^. In a study by Timothy S. Blackwell et al., they observed a significant reduction in MPE formation in mice lacking IL-5. This reduction coincided with decreased levels of eosinophils and myeloid-derived suppressor cells (MDSCs)^26^. Conversely, our investigation, using Eos-null mice, demonstrated that eosinophils inhibited MPE formation. IL-5, known for its role in promoting eosinophil development and allergic responses, also influences B cells, MDSCs and neutrophils^31,32^. Studies show that IL-5 expression in specific cells can induce massive eosinophilia and an increase in total white blood cells^30^. This discrepancy underscores the complexity of IL-5 mechanism in experimental MPE formation, potentially involving eosinophil recruitment, MDSCs influence, or other facets of IL-5 signaling. Understanding the intricate role of IL-5, eosinophils, and MDSCs in MPE formation remains a complex field requiring further investigation.

MPE is mainly the effect of tumor metastasis. Actually, eosinophils exhibit a diverse impact on tumor metastasis. In several studies, eosinophilic infiltration demonstrated promising prognostic value in certain cancers. Gastric cancer showed improved overall survival with eosinophilic infiltration^33^. Human colonic carcinomas displayed higher survival rates and fewer metastases with elevated eosinophil counts^34^. Non-metastatic carcinoma patients exhibited increased eosinophil numbers compared to metastatic carcinoma patients^35^. Additionally, lung cancer patients with EPE have better prognoses than non-EPE patients^27^. Furthermore, other research highlights the potential antitumor effects of eosinophils in the tumor metastasis. In the context of pulmonary metastatic mammary tumors, eosinophils inhibited tumor growth by releasing EPX and effectively killing breast tumor cells (e.g., EO771 cells)^36^. Ariel Munitz et al. discovered that eosinophils affected by breast cancer lung metastases boosted antitumor immunity by facilitating the infiltration of CD4^+^T and CD8^+^T cells promoting an immune response against tumors^21^. However, our prior findings suggesting a possible exacerbation of tumor metastasis due to eosinophilic inflammation, which may be mediated through direct cross-talk between eosinophils and tumor cells via the CLL6-CCR1 axis^25^. Overall, the role of eosinophils during tumor metastasis is likely determined by the tumor type, metastatic site, and the microenvironment. For example, metastasis-entrained eosinophils and inflammation-entrained eosinophils in the lung may display different features and functions, which is crucial for developing targeted therapies and interventions in cancer treatment.

The role of eosinophils in the tumor microenvironment encompasses both pro-tumor and anti-tumor activities^20^. Recent understanding highlights the plasticity of immune cells^37,38^, suggesting varied phenotypes among eosinophils^18,39–42^, leading to distinct responses based on the tumor context. Eosinophils seem to react differently to various mediators, including IFN-γ, IL-5, IL-33, CCL11^24,43–46^, and tumor-derived TSLP^47^, resulting in either enhanced cancer cell killing or promotion of tumor growth. Our RNA-Sequencing data revealed eosinophils’ ability to respond to upstream IFN-γ, TNF-α, IL-21, and IFN-α/β signaling, which could be secreted by CD4^+^T cells and CD8^+^T cells. The predicted upstream transcription regulators IRF3 and IRF7 were reported to protect against infections and cancer^48,49^. Furthermore, another predicted upstream transcription regulators TP53, has previously been shown to reduce the expression of M2 transcripts in macrophages^50^. Surprisingly, their transcriptome closely resembles metastasis-entrained eosinophils^21^, hinting at potential anti-tumor properties in the MPE environment. Additionally, the absence of eosinophils appears to heighten immunosuppression within MPE, suggesting their role in modulating several immune cells. Better understanding of the eosinophil–lymphocyte interactions in the tumor microenvironment might encourage the development and optimization of therapeutic strategies for cancer immunotherapy^51^. Thus, further exploration is needed to decipher how eosinophils influence NK cells and T cell subsets, which will contribute to the advancement of therapeutic strategies for managing MPE.

Multiple clinical observations link eosinophils to cancer immunotherapy^22,46^. Given the transformative impact of gene therapy and immunotherapy on MPE treatment^52^, our research highlights the potential of adaptive eosinophil transfer and strategies boosting eosinophil levels as a step forward in developing eosinophil-based immunotherapies to target MPE.

In summary, the eosinophils recruitment mediated by CCL11 and CCR3 exerts inhibitory effects on the formation of MPE by reshaping its microenvironment probably. These findings present a promising avenue for exploring the potential utilization of eosinophils in clinical strategies aimed at managing MPE.

## Methods

### Human subjects

Patients confirmed MPE and benign pleural effusions (BPE) admitted between February 13, 2022 to October 31, 2023 were recruited (Supplementary Table 1). MPE, BPE, and blood samples from each patient were collected at the Second Affiliated Hospital of Zhejiang University School of Medicine, the Shanghai General Hospital of Shanghai Jiao Tong University School of Medicine, and the Union Hospital of Huazhong University of Science and Technology of Tongji Medical College. At the time of sample collection, the patients had not received any therapy, like anti-cancer therapy, corticosteroids, or other non-steroidal anti-inflammatory drugs. All procedures performed in studies involving human participants were in accordance with the ethical standards of the ethics committees of the Second Affiliated Hospital of Zhejiang University School of Medicine, the Shanghai General Hospital of Shanghai Jiao Tong University School of Medicine, and the Union Hospital of Huazhong University of Science and Technology of Tongji Medical College institutions (approval number: 2022 NO.0119, 2022 NO.167, and [2022] IEC [252], respectively) and with the 1964 Helsinki Declaration and its later amendments or comparable ethical standards. Prior to participation in the study, all patients were informed and signed an informed consent form.

### Mice

C57 BL/6, BALB/C Wild-type (WT) mice and CD45 locus (B6-CD45.1) mice were acquired from Shanghai SLAC Laboratory Animal Co. Ltd. (Shanghai, China). Eosinophil peroxidase-promoter diphtheria toxin A chain transgenic (eosinophil-deficient [Eos-null]) mice^29^ and Cd3δ -promoter *Il5* transgenic (*Il5* Tg) mice^30^ were generated as previously described and provided by the late Dr. James J. Lee (Department of Biochemistry and Molecular Biology, Mayo Clinic, Rochester, Minnesota, USA). Genotypes of transgenic mice and WT littermates were confirmed through polymerase chain reaction analysis of tail snip DNA. All mice were 6- to 8-week-old, sex- and weight-matched, and randomly assigned to different groups. Mice were housed in a specific pathogen-free facility in the Zhejiang University animal center and received food and water ad libitum. Animal experiments were conducted in strict accordance with the protocols approved by the Ethics Committee for Animal Studies at Zhejiang University (approval number: ZJU20220030).

### Syngeneic mouse MPE and myocardial infarction (MI) models

MPE models were prepared by intrapleural injection of 1.5 × 10^5^ LLC, LLC-Luci, MC38, or 4T1 cells as previously described^53^. See the Supplementary Materials Methods for details about the cell lines. Briefly, mice were anesthetized by the intraperitoneal injection (i.p.) of sodium pentobarbital (40 mg/kg), and the area overlying the anterior and lateral chest wall was disinfected. Following that, a 1 cm wide skin incision was made at the xiphoid level on the left anterolateral thoracic area. Fascia and muscle were removed until the ribs and pleural cavity were visible, allowing 50 μL of cell suspension (3 × 10^6^ cells/mL) to be injected easily through an intercostal space. A 5–0 monofilament silk suture was used to close the incision. Animals were hydrated with 1 mL (i.p.) of normal saline and monitored until they recovered. A unique technique previously described was used to cause MI by permanently clamping the left anterior descending (LAD) coronary artery for the control of the MPE model^54,55^. Ischemia was confirmed by distal myocardial bleaching and the identical process was performed on sham-operated mice without LAD ligation. Mice were sacrificed at different days (0, 2, 3, 4, 6, 8, 9, 10, 11, 12, 14) post-MPE or post-MI by the sodium pentobarbital (70 mg/kg) (i.p.), and the blood sampled were collected by eyeball extirpating for further studies. Before harvest, an echocardiogram was performed and mouse tibia length was measured for MI mice. For pleural lavage, 1 mL of phosphate-buffered saline (PBS) was injected intrapleurally and withdrawn after a 30-second wait^26,56–58^. Mice were sacrificed when moribund (11–14 days post-tumor cell injection) for survival, pleural fluid, pleural tumor, and pleural permeability analyses. In survival experiments, a > 20% decline in body weight compared to initial weight or death events indicated biological death. Regarding pleural fluid, mice with volumes ≥100 μL were considered to have MPE and underwent fluid aspiration, while those with volumes <100 μL were deemed pleural effusion-free and underwent pleural lavage. Total MPE nucleated cells were counted using automatic cell counter.

### Intervention for the MPE model

Recombinant mouse C-C motif chemokine ligand 11 (rmCCL11) (0.5 μg/mice in 100 μL PBS) (PeProTech, Cat#250-01) or PBS (100 μL) (Hyclone, Cat#SH30256.01) was administered intrapleurally every 3 days starting from day 4. Small molecule C-C motif chemokine receptor 3 (CCR3) inhibitors SB297006 (Selleck, Cat#S0129) and corn oil (Solarbio LIFE SCIENCE, Cat#8001-30-7) as vehicle were delivered intraperitoneally at a dose of 5 mg/kg every 12 h. Peripheral blood (PB) and spleen eosinophils from *Il5* Tg mice were resuspended in 200 μL of PBS and injected into the tail vein (5–10 × 10^6^ eosinophils/per mouse). See the Supplementary Materials Methods for details on the isolation of eosinophils.

### Flow cytometry (FC) analysis

To prevent non-specific binding, cells were incubated with purified anti-mouse CD16/32 monoclonal antibodies (Biolegend (1 in 5 dilution), Cat#101320) for 15 min at room temperature. Next, cells were incubated for 30 min on ice in the dark with appropriate dilutions of various combinations of anti-mouse cell surface antibodies. The following antibodies were used for FC analysis. For eosinophils: Brilliant Violet 605-CD45 (Biolegend (1 in 200 dilution), Cat#103155), APC/Cy7-CD11b (Biolegend (1 in 200 dilution), Cat#101226), APC-CD11c (Biolegend (1 in 200 dilution), Cat#117310), PE/Cy7-Gr1 (Biolegend (1 in 200 dilution), Cat# 108416), FITC-F4/80 (Biolegend (1 in 200 dilution), Cat#123107), PE/Cy7-F4/80 (Biolegend (1 in 200 dilution), Cat#123114), PE-SiglecF (BD Biosciences (1 in 100 dilution), Cat#552126), APC-CCR3 (Biolegend (1 in 200 dilution), Cat#144511), and FITC-CD45.2 (Biolegend (1 in 200 dilution), Cat#109805).

For FC analysis of human eosinophils in pleural effusions, the following antibodies (all purchased from Biolegend) were used: FITC-CD45 (1 in 50 dilution, Cat#368507), APC-CCR3 (1 in 50 dilution, Cat#310707), and PE-Siglec8 (1 in 50 dilution, Cat# 347103).

All data were acquired using a CytoFlex analyzer (Beckman Coulter Life Science) and analyzed with FlowJo 10.8.1 (BD Biosciences) or CytExpert 2.0 software (Beckman Coulter Life Science). Doublets and dead cells were excluded based on forward and side scatter and DAPI (1 in 1000 dilution) following the manufacturer’s instructions.

### Bioluminescence imaging

MPE models were prepared by intrapleural injection of LLC-Luc cells (1.5 × 10^5^ per mouse). On day 14, MPE mice were anesthetized with 1% pentobarbital sodium and received an intraperitoneal injection of D-luciferin (Selleck, Cat#S7763) following the manufacturer’s instructions. After 10 min, mice were imaged with a 1-min exposure time using the IVIS Lumina III system (PerkinElmer). Data were analyzed using Living Image® v.4.3.1 (Perkin-Elmer).

### Pleural permeability assay

Mice with MPE received an intravenous injection of 0.1 mL of Evans Blue (10 mg/mL) (Thermo Fisher Scientific, Cat#A16774.18) and were sacrificed after 30 min. The concentration of Evans Blue in the pleural fluid was determined by measuring absorbance at a wavelength of 630 nm using a microplate reader (Tecan)^59^.

### Statistical analyses

All data were included in the experiments. Data are presented as mean ± SEM. Statistical analysis was performed using Prism 9.0 (GraphPad Software) and R. For two-group comparisons, *P* values were calculated using unpaired or paired two-tailed Student’s *t* tests, unless specified otherwise. Comparing more than two groups involved one-way and two-way ANOVA with Bonferroni tests, and Kaplan–Meier survival estimates utilized the log-rank test when appropriate. *P* < 0.05 indicated statistically significant differences.

### Reporting summary

Further information on research design is available in the Nature Research Reporting Summary linked to this article.

### Supplementary information

Supplementary Materials

Reporting summary

## Data Availability

The RNA-seq data generated in this study were deposited in the GEO under the accession number GSE245575. CyTOF data have been deposited with the FlowRepository (FR-FCM-Z759). Additional data of this study are available from the corresponding author upon request.

## Code availability

The underlying code for this study is available in CRAN and Bioconductor, and can be accessed via this link: https://cran.r-project.org and https://www.bioconductor.org.

## References

[1] Semaan R, Feller-Kopman D, Slatore C, Sockrider M (2016). Malignant pleural effusions. Am. J. Respir. Crit. Care Med..

[2] Psallidas I, Kalomenidis I, Porcel JM, Robinson BW, Stathopoulos GT (2016). Malignant pleural effusion: from bench to bedside. Eur. Respir. Rev..

[3] Turajlic S, Swanton C (2016). Metastasis as an evolutionary process. Science.

[4] Stathopoulos GT, Kalomenidis I (2012). Malignant pleural effusion. Am. J. Respir. Crit. Care.

[5] Burgers JA (2008). Pleural drainage and pleurodesis: implementation of guidelines in four hospitals. Eur. Respir. J..

[6] Rintoul RC (2014). Efficacy and cost of video-assisted thoracoscopic partial pleurectomy versus talc pleurodesis in patients with malignant pleural mesothelioma (MesoVATS): an open-label, randomised, controlled trial. Lancet.

[7] Walker S, Mercer R, Maskell N, Rahman NM (2020). Malignant pleural effusion management: keeping the flood gates shut. Lancet Respir. Med..

[8] Murthy P (2019). Making cold malignant pleural effusions hot: driving novel immunotherapies. Oncoimmunology.

[9] Horton B, Spranger S (2018). A tumor cell-intrinsic Yin-Yang determining immune evasion. Immunity.

[10] Spranger S, Gajewski TF (2018). Impact of oncogenic pathways on evasion of antitumour immune responses. Nat. Rev. Cancer.

[11] Andre F (2002). Malignant effusions and immunogenic tumour-derived exosomes. Lancet.

[12] Vignali, P. D. A. et al. Hypoxia drives CD39-dependent suppressor function in exhausted T cells to limit antitumor immunity. *Nat. Immunol.* (2022). 10.1038/s41590-022-01379-9 PMC10402660 36543958

[13] Li XY (2021). Intrapleural injection of Anti-PD1 antibody: a novel management of malignant pleural effusion. Front. Immunol..

[14] Adusumilli PS (2014). Regional delivery of mesothelin-targeted CAR T cell therapy generates potent and long-lasting CD4-dependent tumor immunity. Sci. Transl. Med..

[15] Stacy NI, Ackerman SJ (2021). A tribute to eosinophils from a comparative and evolutionary perspective. J. Allergy Clin. Immunol..

[16] Shah K, Ignacio A, McCoy KD, Harris NL (2020). The emerging roles of eosinophils in mucosal homeostasis. Mucosal Immunol..

[17] Klion AD, Ackerman SJ, Bochner BS (2020). Contributions of eosinophils to human health and disease. Annu. Rev. Pathol-Mech..

[18] Weller PF, Spencer LA (2017). Functions of tissue-resident eosinophils. Nat. Rev. Immunol..

[19] Rosenberg HF, Dyer KD, Foster PS (2013). Eosinophils: changing perspectives in health and disease. Nat. Rev. Immunol..

[20] Grisaru-Tal S, Itan M, Klion AD, Munitz A (2020). A new dawn for eosinophils in the tumour microenvironment. Nat. Rev. Cancer.

[21] Grisaru-Tal S (2021). Metastasis-entrained eosinophils enhance lymphocyte-mediated antitumor immunity. Cancer Res..

[22] Lai W (2021). Human pluripotent stem cell-derived eosinophils reveal potent cytotoxicity against solid tumors. Stem Cell Rep..

[23] Arnold IC (2020). The GM-CSF-IRF5 signaling axis in eosinophils promotes antitumor immunity through activation of type 1 T cell responses. J. Exp. Med..

[24] Reichman H (2019). Activated eosinophils exert antitumorigenic activities in colorectal cancer. Cancer Immunol. Res..

[25] Li F (2021). Eosinophilic inflammation promotes CCL6-dependent metastatic tumor growth. Sci. Adv..

[26] Stathopoulos GT (2010). Host-derived interleukin-5 promotes adenocarcinoma-induced malignant pleural effusion. Am. J. Respir. Crit. Care Med..

[27] Takeuchi E (2022). Eosinophilic pleural effusion due to lung cancer has a better prognosis than non-eosinophilic malignant pleural effusion. Cancer Immunol. Immun..

[28] Clive AO (2014). Predicting survival in malignant pleural effusion: development and validation of the LENT prognostic score. Thorax.

[29] Lee JJ (2004). Defining a link with asthma in mice congenitally deficient in eosinophils. Science.

[30] Lee NA (1997). Expression of IL-5 in thymocytes T cells leads to the development of a massive eosinophilia, extramedullary eosinophilopoiesis, and unique histopathologies. J. Immunol..

[31] Dougan M, Dranoff G, Dougan SK (2019). GM-CSF, IL-3, and IL-5 Family of Cytokines: Regulators of Inflammation. Immunity.

[32] Huffnagle GB, Boyd MB, Street NE, Lipscomb MF (1998). IL-5 is required for eosinophil recruitment, crystal deposition, and mononuclear cell recruitment during a pulmonary infection in genetically susceptible mice (C57BL/6). J. Immunol..

[33] Songun, I. et al. Expression of oncoproteins and the amount of eosinophilic and lymphocytic infiltrates can be used as prognostic factors in gastric cancer. Dutch Gastric Cancer Group (DGCG). *Br. J. Cancer* 74, 1783–1788 (1996). 10.1038/bjc.1996.630 PMC2077204 8956793

[34] Pretlow TP (1983). Eosinophil infiltration of human colonic carcinomas as a prognostic indicator. Cancer Res..

[35] Jain M (2014). Assessment of tissue eosinophilia as a prognosticator in oral epithelial dysplasia and oral squamous cell carcinoma-an image analysis study. Pathol. Res. Int..

[36] Cederberg RA (2022). Eosinophils decrease pulmonary metastatic mammary tumor growth. Front. Oncol..

[37] Zhu J, Paul WE (2010). Heterogeneity and plasticity of T helper cells. Cell Res..

[38] Ricketts TD, Prieto-Dominguez N, Gowda PS, Ubil E (2021). Mechanisms of macrophage plasticity in the tumor environment: manipulating activation state to improve outcomes. Front. Immunol..

[39] Marichal T, Mesnil C, Bureau F (2017). Homeostatic Eosinophils: characteristics and functions. Front. Med..

[40] Mesnil C (2016). Lung-resident eosinophils represent a distinct regulatory eosinophil subset. J. Clin. Investig..

[41] Zhu C (2020). Homeostatic and early-recruited CD101(-) eosinophils suppress endotoxin-induced acute lung injury. Eur. Respir. J..

[42] Dolitzky A (2021). Transcriptional profiling of mouse eosinophils identifies distinct gene signatures following cellular activation. Front. Immunol..

[43] Simson L (2007). Regulation of carcinogenesis by IL-5 and CCL11: a potential role for eosinophils in tumor immune surveillance. J. Immunol..

[44] Kataoka S, Konishi Y, Nishio Y, Fujikawa-Adachi K, Tominaga A (2004). Antitumor activity of eosinophils activated by IL-5 and eotaxin against hepatocellular carcinoma. DNA Cell Biol..

[45] Lucarini V (2017). IL-33 restricts tumor growth and inhibits pulmonary metastasis in melanoma-bearing mice through eosinophils. Oncoimmunology.

[46] Hollande C (2019). Inhibition of the dipeptidyl peptidase DPP4 (CD26) reveals IL-33-dependent eosinophil-mediated control of tumor growth. Nat. Immunol..

[47] Xie F (2015). The infiltration and functional regulation of eosinophils induced by TSLP promote the proliferation of cervical cancer cell. Cancer Lett..

[48] King KR (2017). IRF3 and type I interferons fuel a fatal response to myocardial infarction. Nat. Med..

[49] Zilionis R (2019). Single-cell transcriptomics of human and mouse lung cancers reveals conserved myeloid populations across individuals and species. Immunity.

[50] Li L (2015). A unique role for p53 in the regulation of M2 macrophage polarization. Cell Death Differ..

[51] Grisaru-Tal S, Rothenberg ME, Munitz A (2022). Eosinophil-lymphocyte interactions in the tumor microenvironment and cancer immunotherapy. Nat. Immunol..

[52] Sterman DH (2016). Pilot and feasibility trial evaluating immuno-gene therapy of malignant mesothelioma using intrapleural delivery of Adenovirus-IFNα combined with chemotherapy. Clin. Cancer Res..

[53] Stathopoulos GT, Kalomenidis I (2009). Animal models of malignant pleural effusion. Curr. Opin. Pulm Med..

[54] Ma X (2019). A mouse model of heart failure exhibiting pulmonary edema and pleural effusion: Useful for testing new drugs. J. Pharmacol. Toxicol. Methods.

[55] Gao E (2010). A novel and efficient model of coronary artery ligation and myocardial infarction in the mouse. Circ. Res..

[56] Stathopoulos GT (2008). A central role for tumor-derived monocyte chemoattractant protein-1 in malignant pleural effusion. J. Natl Cancer Inst..

[57] Marazioti A (2013). Beneficial impact of CCL2 and CCL12 neutralization on experimental malignant pleural effusion. PLoS One.

[58] Stathopoulos GT (2007). Tumor necrosis factor-alpha promotes malignant pleural effusion. Cancer Res..

[59] Lin H (2014). Interplay of Th1 and Th17 cells in murine models of malignant pleural effusion. Am. J. Respir. Crit. Care Med..

