# Supplementary material for: CCL11/CCR3-dependent eosinophilia alleviates malignant pleural effusions and improves prognosis
Source: NPJ Precis Oncol. 2024 Jun 29;8:138. doi: 10.1038/s41698-024-00608-8 (PMC11217290; doi:10.1038/s41698-024-00608-8)
Supplement: Supplementary file 1 — Supplementary Materials [file 41698_2024_608_MOESM1_ESM.pdf]

Supplementary File for

CCL11/CCR3-dependent eosinophilia alleviates malignant pleural effusions and improves prognosis

Min Zhang[1*], Lixia Xia[1*], Wenbei Peng[6*], Guogang Xie[5*], Fei Li[1*], Chao Zhang[1], Madiha Zahra Syeda[1], Yue Hu[1], Fen Lan[1], Fugui Yan[1], Zhangchu Jin[1], Xufei Du[1], Yinling Han[1], Baihui Lv[1], Yuejue Wang[1], Miao Li[1], Xia Fei[1], Yun Zhao[1], Kaijun Chen[1], Yan Chen[2], Wen Li[1], Zhihua Chen[1], Qiong Zhou[6†], Min Zhang[5†], Songmin Ying[1,2,3†], Huahao Shen[1,4†]

This file includes:

Supplementary Methods

Supplementary Figures

Supplementary Tables

Supplementary References

Supplementary Methods

Cell lines and cell culture

Mouse LLC, MC38, 4T1, and LLC-Luc[1] cells were cultured in Dulbecco's modified Eagle's medium (DMEM) (Hyclone; Cat#SH30023.01) with 10% fetal bovine serum (FBS) (Gibco; Cat#10437028) and penicillin-streptomycin (100 U/mL) (Thermo Fisher; Cat#15140122). The cells were incubated at 37°C in a 5% CO_2 humidified atmosphere. Cell lines were biannually tested for identity (short tandem repeat method) and checked for Mycoplasma Spp. (PCR) every 2 weeks.

Preparation of LLC cell-conditioned media without fetal bovine serum

LLC cell-conditioned media was prepared by culturing confluent LLC tumor cells in serum-free media for 72 hours, followed by centrifugation to remove residual cells and debris[2].

Echocardiography

The echocardiogram was used to select the post-MI mice. Mice were given isoflurane anesthesia, and the echocardiography equipment (VINNO, VINNO D6VET, China), which has a 23-MHz transducer, was used to do the echocardiographic evaluation. At different days after MI, two-dimensional echocardiographic images of the parasternal long axis M-mode were acquired.

The left ventricle (LV) wall thickness, end-diastolic diameter (LV EDD), and end-systolic diameter (LV ESD) were measured using M-mode tracings acquired at the mid-papillary muscle level. The built-in software program determined the percentage of fraction shortening (%FS), ejection fraction (%EF), and other cardiac functions. The VINNO software was used for echocardiography analysis. Finally, choose the post-MI mice with impaired cardiac function for the future analysis.

Isolation of eosinophils.

For migration, cell killing assessment and adaptive transfer： Eosinophils were isolated from *Il5* Tg mouse PB and spleen using MACS with percoll (GE healthcare; Cat#65455-52-9) gradient, biotin anti-mouse CD4 (Biolegend (0.2 µg per 10^6 cells in 100 µL volume); Cat#100404), CD8a (Biolegend (0.2 µg per 10^6 cells in 100 µL volume); Cat#100704), B220 (Biolegend (0.2 µg per 10^6 cells in 100 µL volume); Cat#103204), NK1.1 (Biolegend (0.2 µg per 10^6 cells in 100 µL volume); Cat#108704), Ly6G (Biolegend (0.2 µg per 10^6 cells in 100 µL volume); Cat# 127603), CD115 (Biolegend (0.2 µg per 10^6 cells in 100 µL volume); Cat#135507), CD11c (Biolegend (0.2 µg per 10^6 cells in 100 µL volume); Cat#117303) and Ter119 (Biolegend (0.2 µg per 10^6 cells in 100 µL volume); Cat#116204) antibodies, and anti-biotin magnetic beads (Miltenyi Biotec; Cat#130-090-485). Eosinophil purity was confirmed as >95%

(viability >98%) via FC analysis. **For RNA sequencing:** Eosinophils were isolated from LLC MPE-bearing WT mouse bone marrow (BM) and pleural cavity (PC) using MACS and fluorescence-activated cell sorting (FACS). Red blood cells (RBC) were removed. The bone marrow cells were enriched with biotin-F4/80 (Biolegend (1 in 200 dilution), Cat#123106) antibody and anti-biotin magnetic beads for positive isolation, while the cells in the pleural cavity were enriched with biotin-CD45 (Biolegend (1 in 200 dilution), Cat#103103). Eosinophils were sorted as $DAPI^{-}CD45^{+}SiglecF^{+}SSC^{hi}$ (bone marrow) and $DAPI^{-}CD11C^{-}Gr1^{-/low}CD11b^{+}F4/80^{+}SiglecF^{+}SSC^{hi}$ (pleural cavity) with a FACSAria instrument (BD Biosciences), with a purity of >99% (viability >99%). The purification of eosinophils was identified by Wright-Giemsa staining (Baso, Cat#BA-4017).

Eosinophil migration assay

We tested eosinophil migration using Transwell chambers with 5-μm pore filters (Merck, Cat#CLS3421)[2]. 10^6/mL eosinophils per 400μL RPMI-1640 medium were seeded in the upper chamber, 10% LLC tumor cell-conditioned media, and 10% pleural lavage fluid in the lower chamber. After 12 hours, we counted the eosinophils using a flow cytometer and cell counter.

Cell killing assessment

Non-contact co-culture of target tumor cells with eosinophils

We evaluated eosinophil non-contact killing using Transwell chambers containing 4-μm pore filters (Merck, Cat#CLS3413). LLC, 4T1, and MC38 cells were seeded in the lower chamber, reaching a final count of $5×10^5$ cells per well. Eosinophils, suspended in 400μL RPMI-1640 medium, were seeded in the upper chamber at different ratios (0:1, 1:1, 2:1, 5:1) in relation to tumor cells. Following a 4-hour incubation, DAPI staining was swiftly applied to detect tumor cells and identify dead cells within a 5-minute timeframe according to the manual.

Contact co-culture of target tumor cells with eosinophils

Eosinophils were isolated from the peripheral blood of *Il5*Tg mice. LLC, 4T1, and MC38 cells were pre-treated with CFSE (Thermo Fisher, Cat#C34554) according to the manual and subsequently seeded in 96-well plates to attain a final count of $1×10^4$ cells per well. Then eosinophils were co-seeded with LLC, 4T1, and MC38 cells at different ratios (0:1, 1:1, 2:1, 5:1). After a 4-hour co-culture period, rapid identification and detection of dead cells were conducted using 7-AAD staining (Vazyme, Cat#A213-01) according to the manual, while CFSE staining was utilized to identify tumor cells and eosinophils.

Immunofluorescence

For eosinophil detection, cells on slides were fixed in 4% paraformaldehyde, permeabilized for 20 min in 0.25% Triton X-100/PBS, and blocked for 60 min with bovine serum albumin. Cells were then incubated with primary antibody against eosinophil peroxidase (EPX) (5 μg/mL) (Mayo Clinic) overnight at 4°C. Next, cells were incubated with fluorescent anti-mouse secondary antibody Alexa Fluor®555 (Thermo Fisher Scientific, Cat#A28180) for 1 h for immunofluorescence analysis. 4',6-diamidino-2-phenylindole (DAPI) (Solarbio LIFE SCIENCE (1 in 1000 dilution); Cat# 28718-90-3) was used to stain cell nuclei. For isotype control, primary antibody was omitted. Fluorescence microscopy was performed on an Olympus IX83-FV3000-OSR confocal microscope (Olympus), and images were processed with FV31S-SW Viewer software and FV31S-DT (Ver.2.6) (Olympus).

Immunohistochemistry staining

Immunohistochemistry staining was conducted on 5μm-thick pleural tumor sections to assess Ki67 (Abcam (1 in 1000 dilution), Cat#ab183685), and cleaved-caspase 3 (CST (1 in 200 dilution), Cat#9664) expression. Formalin-fixed, paraffin-embedded samples were prepared on SuperFrost Plus slides (Menzel-Gläser) and processed in an automated autostainer (Bond III; Leica Biosystems) with Bond Polymer Refine Detection and ChromoPlex TM1 Dual

Detection. Diaminobenzidine (Sigma–Aldrich, Cat#D8001) served as the chromogenic substrate. Image J was used for image analysis.

Cytometry by Time-of-Flight (cyTOF)

CyTOF analyses were performed by PLTTech Inc. (Hangzhou, China) according to the previously described protocol[3]. The details are as follows.

CyTOF sample preparation and experimental manipulation

Cell suspensions were collected from pleural lavage as described above. Total cells were stimulated with cell activation cocktail (with Brefeldin A) （Biolegend, Cat# 423303） for 4 h. 4×10^6 cells were washed once with 1xPBS and then stained with 100μL of 250nM cisplatin (Fluidigm) for 5min on ice to exclude dead cells, and then incubated in Fc receptor blocking solution before stained with surface antibodies cocktail for 30 min on ice. Cells were washed twice with FACS buffer (1xPBS + 0.5%BSA) and fixed in 200μL of intercalation solution (Maxpar Fix and Perm Buffer containing 250nM 191/193Ir, Fluidigm) overnight. After fixation, cells were washed once with FACS buffer and then perm buffer, stained with intracellular antibodies cocktail for 30 min on ice. Cells were washed and resuspend with deionized water, adding into 20% EQ beads (Fluidigm), acquired on a mass cytometer (Helios, Fluidigm). The details of the antibodies are shown in **Supplemental Table 2**.

CyTOF data analysis

Data of each sample were debarcoded from raw data using a doublet-filtering scheme[4] with unique mass-tagged barcodes. Each .fcs file generated from different batches were normalized through bead normalization method. Manually gate data using a FlowJo software to exclude to debris, dead cells and doublets, leaving live, single immune cells. Apply the X-shift clustering algorithm[5] to all cells to partition the cells into distinct phenotypes based on marker expression levels. Annotate cell type of each cluster according to its marker expression pattern on a heatmap of cluster vs marker. Use the dimensionality reduction algorithm t-SNE[6] to visualize the high-dimensional data in two dimensions and show distribution of each cluster and marker expression and difference among each group or different sample type. Perform *t*-test statistical analysis on the frequency of annotated cell population.

RNA sequencing and bioinformatics analysis

RNA sequencing: Total RNA was extracted from the eosinophils using RNeasy Micro kit (QIAGEN, Cat#74004) according to the manual instruction. Approximately 40, 000 eosinophils per sample were used for the RNA extraction. Limited RNA（more than 200pg, high-quality） was used for mRNA library construction. The low-input mRNA library was pair-end (100 bp) sequenced on a BGISEQ500 platform (DNBSEQ, BGI-Shenzhen, China). FASTQ files were trimmed using SOAPnuke (v1.4.0) and aligned to the mouse

genome/transcriptome (GENCODE GRCm38 vM25, mm10) using HISAT2 (v2.1.0)[7]. The clean data were mapped to the assembled unique gene by Bowtie2 (v2.2.5)[8]. The expression abundances were estimated (counts and TPM values) using RSEM (v1.2.8)[9].

Bioinformatics analysis: 1) Differential gene analysis and visualization: Differentially expressed genes were identified using DEseq2 in R (P < 0.05, |log2 fold change| > 1) and visualized with Enhanced Volcano package in R[10]. **2) Gene set enrichment analysis:** Performed using R, with annotations from Gene Ontology (GO), Kyoto Encyclopedia of Genes and Genomes (KEGG), and Reactome. Significance thresholds were P <0.05 and |normalized enrichment score| >1 [11]. **3) Upstream regulator analysis (URA):** Using Ingenuity Pathway Analysis (IPA) software identified upstream molecules of differentially expressed genes. Top ten cytokines and transcription factors with significant overlap P-values and activation Z-scores were reported[12].

Cytokine detection

Mouse cytokine array: MPE models in Eos-null (n=3) and WT mice (n=3) were established via intrapleural LLC cell injection (1.5×10^5 cells/per mouse), and pleural lavage samples were collected. Centrifuged samples were examined using a Mouse Cytokine Array GS4000 (RayBiotech, Cat#GSM-CAA-4000-1) following the manufacturer's guidelines. Arrays were imaged with an InnoScan

300 Microarray Scanner, and data were processed and analyzed using GenePix Pro 5.1 and RayBiotech Q-Analyzer programs.

ELISA analysis: Cytokine and chemokine concentrations were measured using ELISA per manufacturer guidelines. Centrifuged pleural lavage, MPE, and peripheral blood supernatant (12,000 rpm, 10 min, 4°C) were analyzed for CCL11 using mouse CCL11 (Biolegend, Cat#443907), human CCL11 (R&D Systems, Cat#DTX00) ELISA kits.

Supplementary Figures

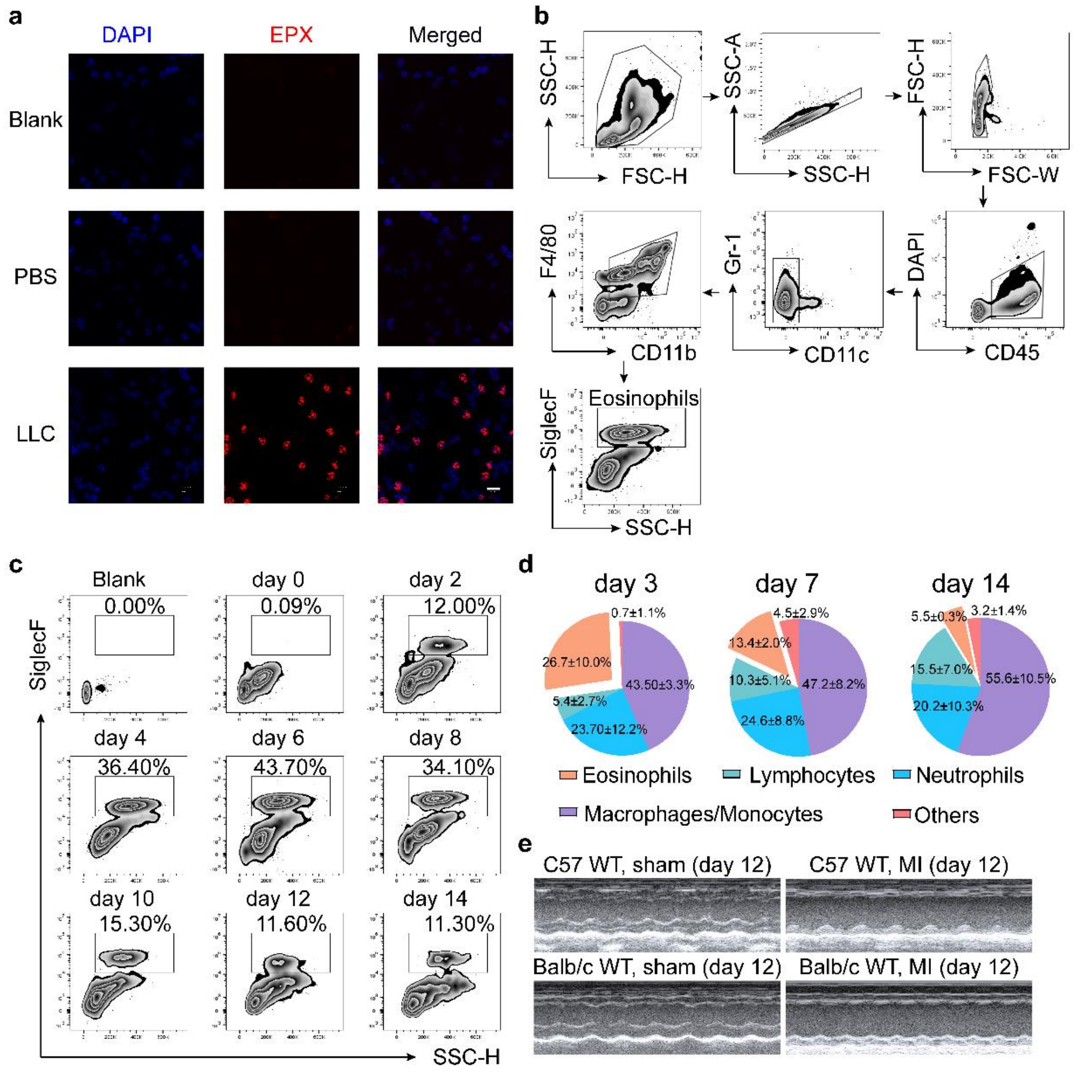

Supplementary Figure 1. Characterization and distribution of eosinophils, and the representative LV M-mode echocardiography images of mice. a. Representative confocal images of eosinophils from pleural lavage fluid at day 3, stained for DAPI and EPX. Scale bar: 20 μm. b. Flow cytometry hierarchical gating strategy for eosinophils in mice. Eosinophils were identified as $DAPI^{-}$ $CD45^{+}Gr1^{-/low}CD11c^{-}CD11b^{+}F4/80^{+}SiglecF^{+}SSC^{hi}$ cells. c. Representative flow cytometry plot of eosinophils in pleural lavage fluid at different days after the

LLC injection. d. Percentage of the pleural cavity's primary leukocyte compartment following LLC injection as determined by a differential count following flinger. e. Representative LV M-mode echocardiography images at day 12 post-MI from different groups of mice. Data presented as mean ± SEM.

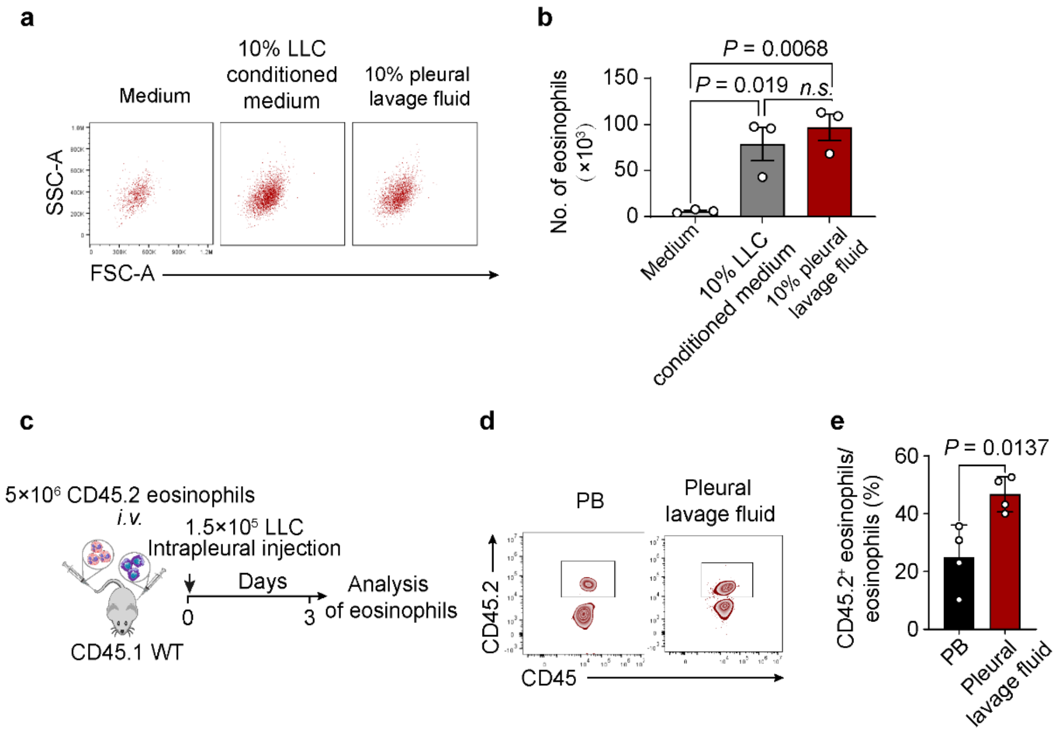

Supplementary Figure 2. Effects of LLC culture supernatants and pleural lavage fluid on eosinophil recruitment. a, b. In vitro eosinophil chemotaxis assay. (a) Representative flow cytometry plot. (b) Eosinophil count in chemotactic chamber's lower layer; n=3. **c-e.** Circulating eosinophils recruited to the pleural cavity. (c) Experimental design schematic. (d) Representative flow cytometry plot. (e) $CD45.2^+$ eosinophils as a percentage of total eosinophils in PB and pleural cavity. Data presented as mean ± SEM. One-way ANOVA with Bonferroni test for b; two-tailed unpaired Student's *t*-test for e. *P*-values <0.05 indicated statistical significance.

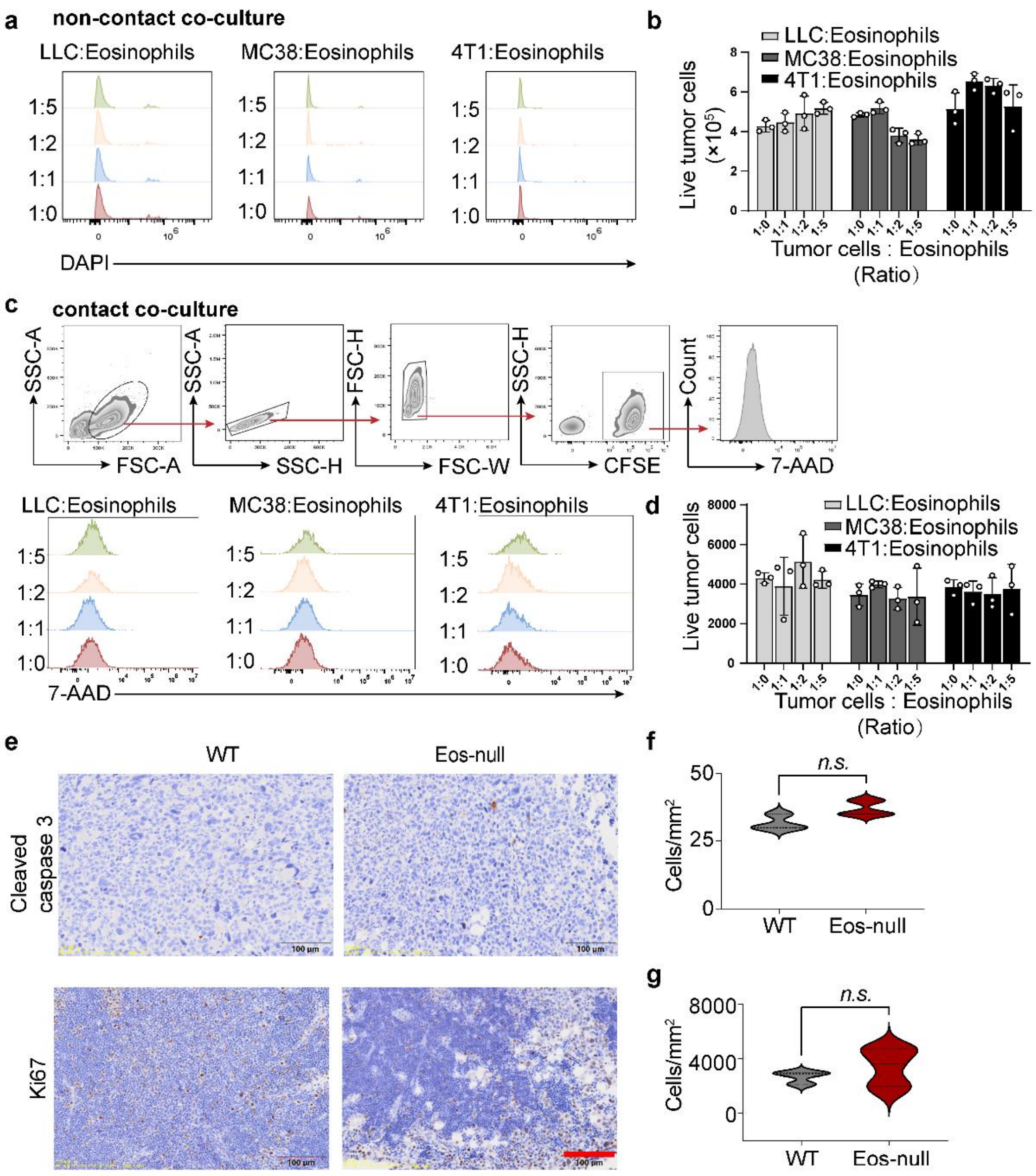

Supplementary Figure 3. Impact of eosinophils' effect on the tumor cells and tumor tissue. a-d. Eosinophils' killing assessment. (a). Representative histograms of DAPI in LLC, 4T1 and MC38 tumor cells for non-contact co-culture of tumor cells and eosinophils at different ratio. (b). The survival of LLC, 4T1 and MC38 tumor cells in non-contact co-culture systerm was determined by FC (n=3). (c). The gating strategy of the contact co-culture system (up). Representative histograms of 7-AAD in LLC, 4T1 and MC38 tumor cells for

contact co-culture of tumor cells and eosinophils at different ratio (down). (d). The survival of LLC, 4T1 and MC38 tumor cells in contact co-culture systerm was determined by FC (n=3). **e-g**. Pleural tumor tissues from WT and Eos-null mice in the LLC-MPE model were analyzed using immunohistochemical staining for cleaved-caspase 3, and Ki-67 (n=5). (e) Representative IHC images for cleaved-caspase 3, and Ki-67 in pleural tumors. Scale: 100 μm. (f) Quantification of cleaved-caspase 3^+ cells per mm2. (g) Quantification of Ki-67^+ cells per mm2. Data presented as mean ± SEM. One-way ANOVA with Bonferroni test for b, c and two-tailed unpaired Student's *t*-test was performed for f, g, h , with *P*-values <0.05 indicating statistical significance.

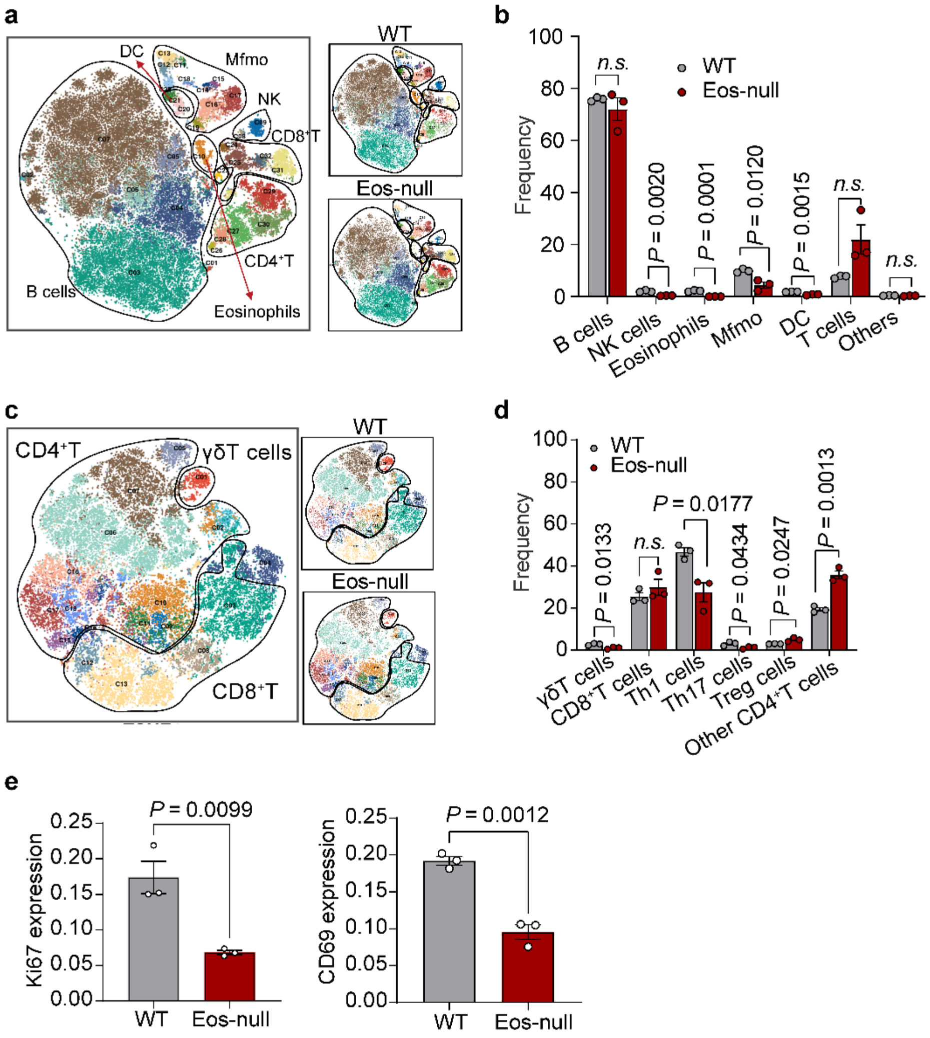

Supplementary Figure 4. Immune cell phenotyping of WT mice and Eos-null mice in the pleural cavity microenvironment after LLC injection. a. Density tSNE plots of $CD45^+$ cells in the pleural cavity from WT (n=3) and Eos-null (n=3) mice. **b.** Frequency of clusters grouped by indicated immune cell subsets. **c.** Density tSNE plots of $CD3^+$ T cells in the pleural cavity from WT (n=3) and Eos-null (n=3) mice. **d.** Frequencies of T cell subtypes in two groups. **e**. The expression of Ki67 and CD69 on $CD3^+$ T cells in two groups. Data were shown as the mean ± SEM. Two-tailed unpaired Student's *t*-test was performed for b, d, e, with *P*-values <0.05 indicating statistical significance.

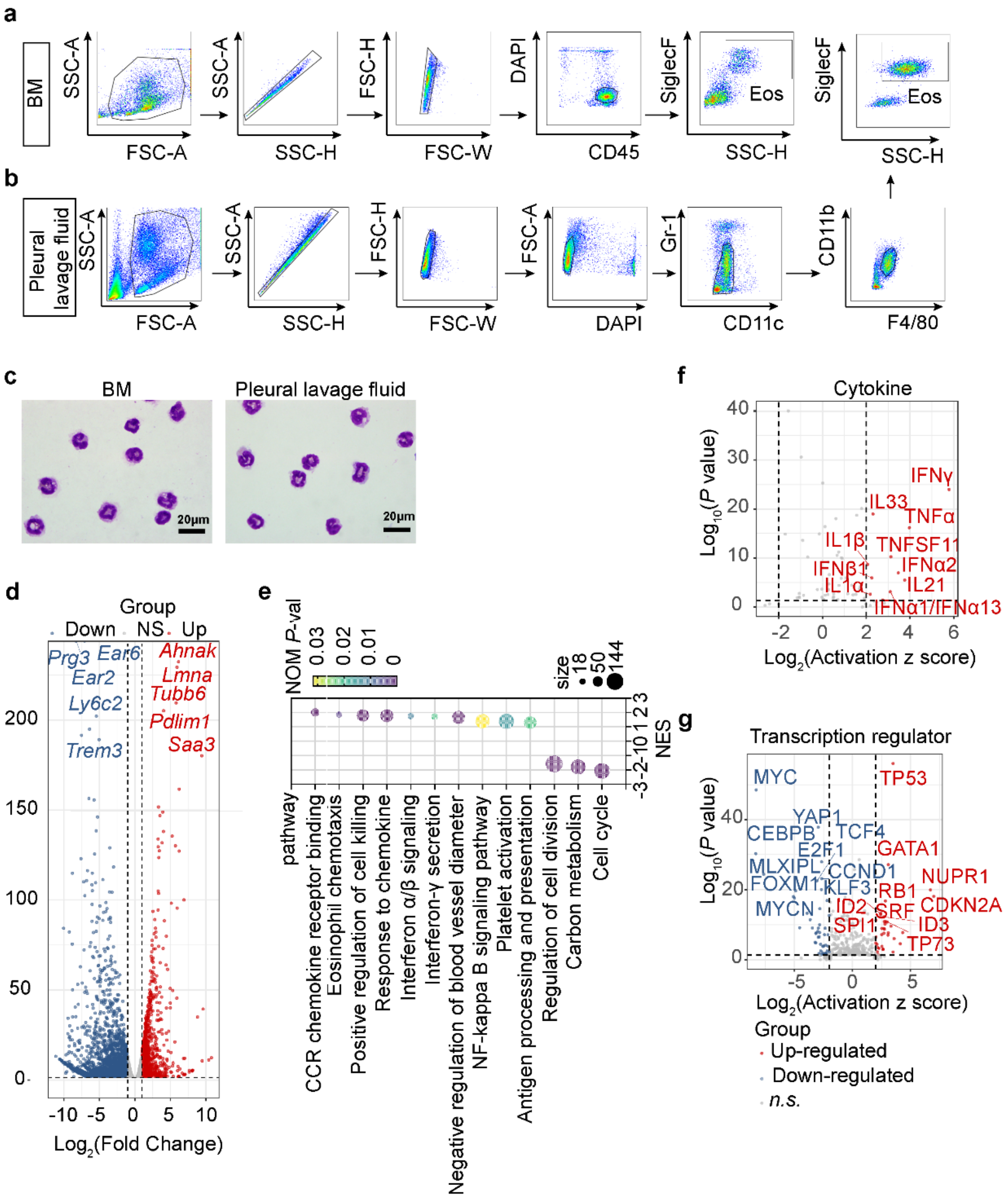

Supplementary Figure 5. Sorting strategies and transcription profiles of eosinophils. a, b, c. Flow cytometry sorting strategy for eosinophils. **(a).** Experimental design schematic: eosinophils isolated from bone marrow and pleural lavage fluid were sorted by MACS and FACS for RNA sequencing**. (b).** Hierarchical gating strategy for eosinophils in BM and pleural cavity using flow

cytometry sorting. **（c）.** Representative images of Wright-Giemsa staining of eosinophils purified from the pleural cavity (left) and bone marrow (right) (n=5 to 10 images per tissue type). Scale bars: 20 μm. **d.** Volcano plot displaying differentially expressed genes (DEGs) between BM and pleural lavage fluid eosinophils. Top five genes labeled. Y-axis *P*-value cutoff: 0.05; x-axis $\log_2$ (fold change) cutoff: ±1.0. **e**. Significant GSEA pathways highlighted. NES: normalized enrichment score; NOM *P*-val: normalized *P*-value; Size: gene count. **f, g.** URA for eosinophil transcriptome. **（f）.** Top 10 upstream regulator cytokines. **（g）.** Top 10 upstream regulator transcription factors.

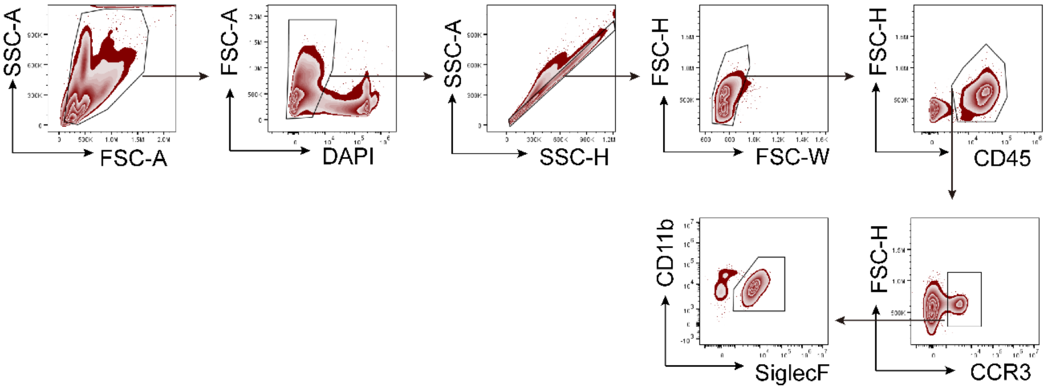

Supplementary Figure 6. Flow cytometry plots displaying CCR3 expression on eosinophils from pleural lavage fluid.

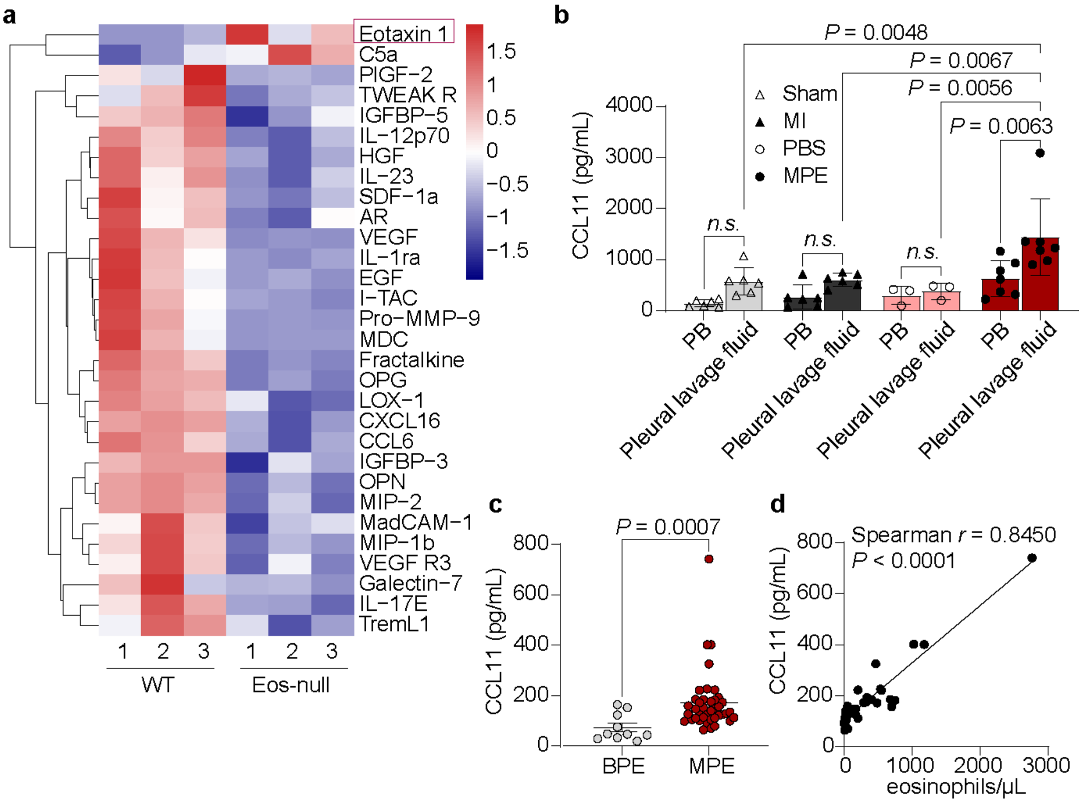

Supplementary Figure 7. CCL11 concentration. a. Heatmap displaying cytokine microarray of pleural lavage fluid supernatant from WT (n=3) and Eos-null mice (n=3). **b.** ELISA measuring CCL11 in the pleural lavage fluid of MPE and MI (n=3-7). **c.** ELISA quantifying CCL11 in human MPE (n=40) and BPE (n=10). **d.** Correlation between eosinophils and CCL11 in humans with MPE (n=40). Data presented as mean ± SEM. Unpaired Student's *t*-test for b, c; Spearman rank correlation coefficients for d. *P*-values <0.05 indicate statistical significance.

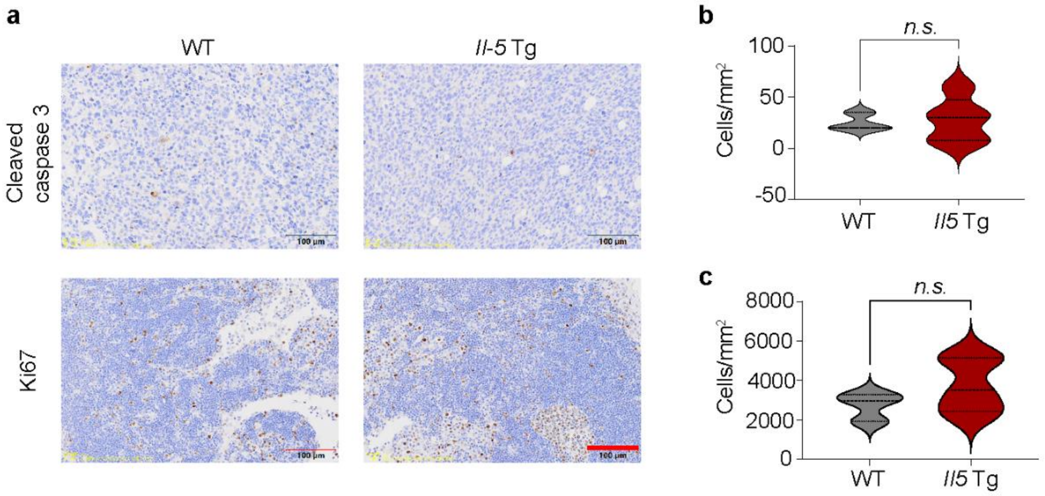

Supplementary Figure 8. Impact of eosinophil overexpression on cleaved-caspase 3 and Ki-67 expression in pleural tumors. **a-c.** Pleural tumor tissues from LLC MPE-bearing model in WT and *Il5* Tg mice were analyzed. Immunohistochemical (IHC) staining for cleaved-caspase 3 and Ki-67 was performed (n=5). **(a).** Representative IHC images of cleaved-caspase 3 and Ki-67 in pleural tumors; scale: 100 μm. **(b).** Quantification of cleaved-caspase 3$^+$ cells per mm^2. **(c).** Quantification of Ki67$^+$ cells per mm^2. Data presented as mean ± SEM. Two-tailed unpaired Student's *t*-test was performed. *P*-values <0.05 indicate statistical significance.

Supplementary Table 1. Basic information of enrolled BPE patients (n=10) and MPE patients (n = 40)

		MPE (n = 40)	BPE (n=10)
Age	Mean age, years (range)	68.8 (55–89)	59 (48-72)
	≥65 (%)	28 (70.0)	3 (30)
	<65 (%)	12 (30.0)	7 (70)
Sex (%)	Male	25 (62.5)	5 (50)
	Female	15 (37.5)	5 (50)
Smoking history (%)	Yes	25 (62.5)	2 (20)
	No	5 (12.5)	8 (80)
	Missing	10 (25.0)	0
pLDH	Mean (SEM), U/L	654.7 (330.0)	300.9 (185.1)
Histologic type (%)	Adenocarcinoma lung cancer	25(65.0)	/
	Squamous lung cancer	4(10.0)	/
	Small cell lung cancer	3 (7.5)	/
	Breast cancer	6 (12.5)	/
	Gynaecological cancer	1 (2.5)	/
	Haematological malignancy	1 (2.5)	/
ECOG PS (%)	0	2 (5)	10 (100)
	1	27 (67.5)	0
	2	5 (12.5)	0
	3	5 (12.5)	0
	4	1 (2.5)	0
NLR (%)	<9	40 (100.0)	10 (100)
	≥9	0 (0.0)	0
Cause (%)	CHF	/	3 (30)
	TPE	/	3 (30)
	Pulmonary embolism	/	2 (20)
	Pneumonia	/	2 (20)

BPE, benign pleural effusion; ECOG PS, Eastern Cooperative Oncology Group (ECOG) Performance Status; NLR, neutrophil tolymphocyte ratio; CHF, congestive heart failure; TPE, tuberculosis pleural effusion.

Supplementary Table 2. Antibodies for cyTOF

Antibodies	metal channel	Clone NO.	Manufacturer	Cat NO.
CD45	89Y	30-F11	Biolegend	103102
CD3ε	115ln	145-2C11	Biolegend	100302
Ki-67	139La	SolA15	eB	14-5698-82
CD103	141Pr	2E7	Biolegend	121402
TCRγ/δ	142Nd	GL3	Biolegend	118101
MHCII（I-A/I-E）	143Nd	M5/114.15.2	Biolegend	107602
CD44	144Nd	IM7	Biolegend	103002
CD80	145Nd	16-10A1	Biolegend	104702
CD279（PD-1）	146Nd	29F.1A12	Biolegend	135202
Ly-6G	147Sm	1A8	Biolegend	127602
CD115（CSF-1R）	148Nd	AFS98	Biolegend	135502
CD19	149Sm	6D5	Biolegend	115502
CD49b（pan-NK cells）	150Nd	DX5	Biolegend	108902
CD335（NKp46）	151Eu	29A1.4	Biolegend	137602
Ly-6C	152Sm	HK1.4	Biolegend	128002
iNOS	153Eu	CXNFT	eB	14-5920-82
CD62L	154Sm	MEL-14	Biolegend	104402
CD11c	155Gd	N418	Biolegend	117302
Gr-1（Ly-6G/Ly-6C）	156Gd	RB6-8C5	Biolegend	108402
IL-4	157Gd	11B11	Biolegend	504102
CD45R（B220）	158Gd	RA3-6B2	Biolegend	103202
F4/80	159Tb	C1：A3-1	BioRAD	MCA497G
CD27	160Gd	LG.3A10	Biolegend	124202
TCRβ chain	161Dy	H57-597	Biolegend	109202
CD69	162Dy	H1.2F3	Biolegend	104502

CD25	163Dy	3C7	Biolegend	101902
CD86	164Dy	GL-1	Biolegend	105002
CD161（NK-1.1）	165Ho	PK136	Biolegend	108702
IL-17A	166Er	TC11-	Biolegend	506902
CD206（MMR）	167Er	C068C2	Biolegend	141702
CD185（CXCR5）	168Er	L138D7	Biolegend	145502
CD127（IL-7Rα）	169Tm	A7R34	Biolegend	135002
T-bet	170Er	4B10	Biolegend	644802
Gata-3	171Yb	TWAJ	eB	14-9966-82
CD192（CCR2）	172Yb	475301	R&D	MAB55381-100
CD278（ICOS）	173Yb	C398.4A	Biolegend	313502
IFN-γ	174Yb	XMG1.2	Bio-Xcell	BE0055
Siglec-F	175Lu	E50-2440	BD	552125
CD366（Tim-3）	176Yb	RMT3-23	Biolegend	119702
CD4	197Au	RM4-5	Biolegend	100520
CD8a	198pt	53-6.7	Biolegend	100716
CD11b	209Bi	M1/70	Biolegend	101202

284

Supplementary Table 3. Marker expression in each sample of cyTOF.

Marker	WT 1	WT 2	WT 3	Eos-null 1	Eos-null 2	Eos-null 3
CD45	2.000	1.914	1.944	2.172	2.127	2.250
CD3e	0.123	0.151	0.153	0.228	0.231	0.378
Ki67	0.602	0.930	0.829	0.440	0.426	0.333
CD103	0.037	0.047	0.039	0.033	0.034	0.057
TCRgd	0.062	0.062	0.055	0.054	0.053	0.044
MHCII	3.266	2.944	3.006	3.088	3.111	2.472
CD44	3.938	4.179	4.050	3.857	3.896	3.055
CD80	0.268	0.407	0.360	0.272	0.289	0.138
PD1	0.064	0.079	0.066	0.062	0.063	0.050
Ly6G	0.010	0.013	0.011	0.009	0.009	0.007
CD115	0.081	0.128	0.107	0.072	0.071	0.042
CD19	2.252	2.433	2.415	2.394	2.327	1.934
CD49b	0.106	0.150	0.124	0.072	0.071	0.055
NKp46	0.046	0.061	0.045	0.020	0.018	0.014
Ly6C	0.349	0.383	0.310	0.283	0.286	0.487
iNOS	0.052	0.091	0.065	0.049	0.062	0.072
CD62L	0.020	0.025	0.022	0.027	0.023	0.046
CD11c	0.154	0.181	0.150	0.079	0.073	0.062

Gr1	0.009	0.013	0.012	0.006	0.006	0.006
IL-4	0.044	0.074	0.055	0.038	0.043	0.044
B220	1.156	0.916	0.831	1.313	1.048	1.341
F4_80	0.637	0.663	0.609	0.389	0.402	0.242
CD27	0.335	0.353	0.325	0.426	0.439	0.787
TCRb	0.301	0.284	0.309	0.612	0.655	1.277
CD69	0.843	0.571	0.609	0.815	0.720	0.712
CD25	0.085	0.128	0.104	0.121	0.111	0.074
CD86	0.667	0.660	0.583	0.498	0.542	0.473
NK1.1	0.089	0.111	0.082	0.032	0.030	0.028
IL_17A	0.047	0.045	0.045	0.041	0.035	0.032
CD206	0.123	0.166	0.138	0.085	0.095	0.047
CXCR5	2.115	2.135	2.149	2.132	1.763	1.365
CD127	0.116	0.110	0.118	0.242	0.248	0.555
Tbet	0.117	0.176	0.124	0.105	0.096	0.126
Gata3	0.032	0.057	0.044	0.031	0.035	0.039
CCR2	0.138	0.174	0.134	0.073	0.073	0.035
ICOS	0.029	0.037	0.036	0.037	0.035	0.049
IFN_γ	0.062	0.098	0.081	0.061	0.064	0.057
SiglecF	0.064	0.082	0.074	0.010	0.010	0.009

Tim3	0.009	0.011	0.009	0.006	0.004	0.005
CD4	0.116	0.117	0.136	0.266	0.277	0.463
CD8a	0.035	0.026	0.030	0.062	0.066	0.192
CD11b	2.139	2.615	2.597	1.713	1.722	1.238

Supplementary Table 4. Cytokines associated with the function of eosinophils measured using the cytokine array

Protein ID	AveExp. WT	AveExp. Eos-null	Log FC	*P*-value	adjusted *P*-value
Eotaxin-2	11.92	11.33	−0.58	0.14	0.59
RANTES	12.24	12.74	0.50	0.49	0.81
IL-4	11.01	11.25	0.24	0.21	0.60
IL-5	10.98	11.17	0.19	0.47	0.81
TSLP	11.66	11.60	−0.06	0.61	0.83
IL-10	10.74	11.03	0.29	0.24	0.66
GM-CSF	10.93	11.11	0.18	0.44	0.81
Granzyme	8.87	8.97	0.10	0.78	0.93
Fas L	9.93	10.13	0.20	0.12	0.56
IFNγ	10.74	10.67	−0.07	0.48	0.81
TNFα	10.64	10.80	0.16	0.50	0.81
IL-33	10.32	10.14	−0.18	0.15	0.60
IL-17	10.93	10.99	0.07	0.52	0.81
IL-21	12.95	12.98	0.03	0.81	0.95
Flt-3L	14.14	14.07	−0.07	0.74	0.91
CD40	10.07	10.23	0.17	0.27	0.69
CD40L	11.32	11.11	−0.21	0.18	0.60
Leptin	8.98	9.50	0.52	0.19	0.60
Leptin R	11.64	11.56	−0.09	0.47	0.81
MMP-2	13.06	13.29	0.23	0.46	0.81

AveExp., average expression; FC, fold change; Flt-3L, fms related receptor tyrosine kinase 3 ligand; GM-CSF, granulocyte-macrophage colony stimulating factor; MMP-2, matrix metallopeptidase 2; RANTES, regulated upon activation,

normally T-expressed, and presumably secreted; TNFα, tumor necrosis factor alpha; TSLP, thymic stromal lymphopoietin.

Supplementary References

1 Wan, C., Sun, Y. J., Tian, Y., Lu, L. S., Dai, X. M. *et al.* Irradiated tumor cell-derived microparticles mediate tumor eradication via cell killing and immune reprogramming. *Sci Adv* **6**, doi:ARTN eaay978910.1126/sciadv.aay9789 (2020).

2 Grisaru, S., Itan, M. & Munitz, A. Analysis of Mouse Eosinophil Migration and Killing of Tumor Cells. *Methods Mol Biol* **2241**, 89-97, doi:10.1007/978-1-0716-1095-4_8 (2021).

3 Han, G. J., Spitzer, M. H., Bendall, S. C., Fantl, W. J. & Nolan, G. P. Metal-isotope-tagged monoclonal antibodies for high-dimensional mass cytometry. *Nat Protoc* **13**, 2121-2148, doi:10.1038/s41596-018-0016-7 (2018).

4 Zunder, E. R., Finck, R., Behbehani, G. K., Amir, E. D., Krishnaswamy, S. *et al.* Palladium-based mass tag cell barcoding with a doublet-filtering scheme and single-cell deconvolution algorithm. *Nat Protoc* **10**, 316-333, doi:10.1038/nprot.2015.020 (2015).

5 Samusik, N., Good, Z., Spitzer, M. H., Davis, K. L. & Nolan, G. P. Automated mapping of phenotype space with single-cell data. *Nat Methods* **13**, 493-+, doi:10.1038/Nmeth.3863 (2016).

6 van der Maaten, L. & Hinton, G. Visualizing Data using t-SNE. *J Mach Learn Res* **9**, 2579-2605 (2008).

7 Kim, D., Langmead, B. & Salzberg, S. L. HISAT: a fast spliced aligner with low memory requirements. *Nat Methods* **12**, 357-360, doi:10.1038/nmeth.3317 (2015).

8 Langmead, B. & Salzberg, S. L. Fast gapped-read alignment with Bowtie 2. *Nat Methods* **9**, 357-359, doi:10.1038/nmeth.1923 (2012).

9 Li, B. & Dewey, C. N. RSEM: accurate transcript quantification from RNA-Seq data with or without a reference genome. *BMC Bioinformatics* **12**, 323, doi:10.1186/1471-2105-12-323 (2011).

10 Love, M. I., Huber, W. & Anders, S. Moderated estimation of fold change and dispersion for RNA-seq data with DESeq2. *Genome Biol* **15**, 550, doi:10.1186/s13059-014-0550-8 (2014).

11 Subramanian, A., Tamayo, P., Mootha, V. K., Mukherjee, S., Ebert, B. L. *et al.* Gene set enrichment analysis: A knowledge-based approach for interpreting genome-wide expression profiles. *P Natl Acad Sci USA* **102**, 15545-15550, doi:10.1073/pnas.0506580102 (2005).

12 Kramer, A., Green, J., Pollard, J., Jr. & Tugendreich, S. Causal analysis approaches in Ingenuity Pathway Analysis. *Bioinformatics* **30**, 523-530, doi:10.1093/bioinformatics/btt703 (2014).
