# Supplementary material for: CCL11/CCR3-dependent eosinophilia alleviates malignant pleural effusions and improves prognosis
Source: NPJ Precis Oncol. 2024 Jun 29;8:138. doi: 10.1038/s41698-024-00608-8 (PMC11217290; doi:10.1038/s41698-024-00608-8)
Supplement: Supplementary file 2 — Reporting summary [file 41698_2024_608_MOESM2_ESM.pdf]

nature portfolio

Corresponding author(s): Huahao Shen

Last updated by author(s): Apr 25, 2024

Reporting Summary

Nature Portfolio wishes to improve the reproducibility of the work that we publish. This form provides structure for consistency and transparency in reporting. For further information on Nature Portfolio policies, see our Editorial Policies and the Editorial Policy Checklist.

Statistics

For all statistical analyses, confirm that the following items are present in the figure legend, table legend, main text, or Methods section.

n/a	Confirmed	
☐	☒	The exact sample size (*n*) for each experimental group/condition, given as a discrete number and unit of measurement
☐	☒	A statement on whether measurements were taken from distinct samples or whether the same sample was measured repeatedly
☐	☒	The statistical test(s) used AND whether they are one- or two-sided<br>*Only common tests should be described solely by name; describe more complex techniques in the Methods section.*
☒	☐	A description of all covariates tested
☒	☐	A description of any assumptions or corrections, such as tests of normality and adjustment for multiple comparisons
☐	☒	A full description of the statistical parameters including central tendency (e.g. means) or other basic estimates (e.g. regression coefficient) AND variation (e.g. standard deviation) or associated estimates of uncertainty (e.g. confidence intervals)
☐	☒	For null hypothesis testing, the test statistic (e.g. *F*, *t*, *r*) with confidence intervals, effect sizes, degrees of freedom and *P* value noted<br>*Give P values as exact values whenever suitable.*
☒	☐	For Bayesian analysis, information on the choice of priors and Markov chain Monte Carlo settings
☒	☐	For hierarchical and complex designs, identification of the appropriate level for tests and full reporting of outcomes
☒	☐	Estimates of effect sizes (e.g. Cohen's *d*, Pearson's *r*), indicating how they were calculated

Our web collection on statistics for biologists contains articles on many of the points above.

Software and code

Policy information about availability of computer code

Data collection	Olympus IX83-FV3000-OSR confocal microscope (Olympus); CytoFlex analyzer (Beckman Coulter Life Science); IVIS Lumina III system (PerkinElmer); BGISEQ500 platform;echocardiography equipment (VINNO, VINNO D6VET, China); mass cytometer (Helios, Fluidigm);GenePix Pro 5.1.
Data analysis	For statistics:Prism 9.0 (GraphPad Software) ; For RNA-Seq R and IPA software; For FC: FlowJo 10.8.1 (BD Biosciences) or CytExpert 2.0 software (Beckman Coulter Life Science); For bioluminescence:Living Image® v.4.3.1 (Perkin-Elmer); For IF:FV31S-SW Viewer software and FV31S-DT (Ver.2.6) (Olympus); For CK array:RayBiotech Q-Analyzer; For IHC:Image J; For cyTOF:1)FlowJo v10.0.7 BD https://www.flowjo.com/ ;2)Normalizer Finck et al., 2013 https://github.com/nolanlab/bead-normalization ;3)Single cell debarcoder Zunder et al., 2015 https://github.com/nolanlab/single-cell-debarcoder ;4)t-SNE van der Maaten and Hinton, 2008 https://github.com/jkrijthe/Rtsne ;5)PARC Stassen et al., 2020 https://github.com/ShobiStassen/PARC ;6)X-shift Samusik et al., 2016 https://github.com/nolanlab/vortex ;7)PhenoGraph Levine et al., 2015 https://github.com/jacoblevine/PhenoGraph

For manuscripts utilizing custom algorithms or software that are central to the research but not yet described in published literature, software must be made available to editors and reviewers. We strongly encourage code deposition in a community repository (e.g. GitHub). See the Nature Portfolio guidelines for submitting code & software for further information.

Data

Policy information about availability of data

All manuscripts must include a data availability statement. This statement should provide the following information, where applicable:

- Accession codes, unique identifiers, or web links for publicly available datasets
- A description of any restrictions on data availability
- For clinical datasets or third party data, please ensure that the statement adheres to our policy

The RNA-seq data generated in this study were deposited in the GEO under the accession number GSE245575. CyTOF data have been deposited with the FlowRepository (FR-FCM-Z759). Additional data of this study are available from the corresponding author upon request.

Research involving human participants, their data, or biological material

Policy information about studies with human participants or human data. See also policy information about sex, gender (identity/presentation), and sexual orientation and race, ethnicity and racism.

Reporting on sex and gender	This study did not conduct sex or gender-based analysis.
Reporting on race, ethnicity, or other socially relevant groupings	This study did not conduct race, ethnicity,or other socially relevant groupings.
Population characteristics	Patients with confirmed MPE patients (n=40) and benign pleural effusions patients (BPE) (n=10) admitted between February 13, 2022 to October 31, 2023 were recruited, with a mean age of 69±8 years (MPE) and 59±8 years (BPE).
Recruitment	Recruited patients had not received any therapy, like anti-cancer therapy, corticosteroids, or other non-steroidal anti-inflammatory drugs.
Ethics oversight	The ethics committees of the Second Affiliated Hospital of Zhejiang University School of Medicine, the Shanghai General Hospital of Shanghai Jiao Tong University School of Medicine, and the Union Hospital of Huazhong University of Science and Technology of Tongji Medical College institutions reviewed and approved the protocol of this study (approval number: 2022 NO.0119, 2022 NO.167, and [2022] IEC [252], respectively). Prior to participation in the study, all patients were informed and signed an informed consent form.

Note that full information on the approval of the study protocol must also be provided in the manuscript.

Field-specific reporting

Please select the one below that is the best fit for your research. If you are not sure, read the appropriate sections before making your selection.

☒ Life sciences ☐ Behavioural & social sciences ☐ Ecological, evolutionary & environmental sciences

For a reference copy of the document with all sections, see nature.com/documents/nr-reporting-summary-flat.pdf

Life sciences study design

All studies must disclose on these points even when the disclosure is negative.

Sample size	No prior sample-size calculation was performed. In all experimental treatment studies, a sample size of 2-15 was used initially. Statistical analysis was performed on the collected data and the results were used to determine if more samples were needed
Data exclusions	No data were excluded from the analyses conducted for this study.
Replication	Each experiment was repeated at least three times.
Randomization	The allocation of samples/organisms/participants into experimental groups was randomized using a computer-generated randomization process.
Blinding	The investigators were blinded to group allocation during both data collection and analysis.

Reporting for specific materials, systems and methods

We require information from authors about some types of materials, experimental systems and methods used in many studies. Here, indicate whether each material, system or method listed is relevant to your study. If you are not sure if a list item applies to your research, read the appropriate section before selecting a response.

Materials & experimental systems

n/a	Involved in the study
☐	☒ Antibodies
☐	☒ Eukaryotic cell lines
☒	☐ Palaeontology and archaeology
☐	☒ Animals and other organisms
☒	☐ Clinical data
☒	☐ Dual use research of concern
☒	☐ Plants

Methods

n/a	Involved in the study
☒	☐ ChIP-seq
☐	☒ Flow cytometry
☒	☐ MRI-based neuroimaging

Antibodies

Antibodies used	IF antibodies:anti-mouse EPX gifted from Jamie J. Lee ;IHC antibodies:Ki67 (Abcam, Cat#ab183685), and cleaved-caspase 3 (CST, Cat#9664) ;FC antibodies: mouse eosinophils: Brilliant Violet 605-CD45 (Biolegend, Cat#103155), APC/Cy7-CD11b (Biolegend, Cat#101226), APC-CD11c (Biolegend, Cat#117310), PE/Cy7-Gr1 (Biolegend, Cat# 108416), FITC-F4/80 (Biolegend, Cat#123107), PE/Cy7-F4/80 (Biolegend, Cat#123114), PE-SiglecF (BD Biosciences, Cat#552126), APC-CCR3 (Biolegend, Cat#144511), and FITC-CD45.2 (Biolegend, Cat#109805).For FC analysis of human eosinophils in pleural effusions, the following antibodies (all purchased from Biolegend) were used: FITC-CD45 (Cat#368507), APC-CCR3 (Cat#310707), and PE-Siglec8 (Cat# 347103). cyTOF antibodies: Data provided in the manuscript.
Validation	All antibodies were well-recognized in the field and have their validation statement on their manufactures' websites.https://www.biolegend.com, https://www.thermofisher.com, https://www.cellsignal.com, https://bxcell.com, https://www.bdbiosciences.com/en-us, https:// www.sinobiological.com, or http://www.abmole.com. These antibodies are further validated for species and application, and are routinely used in our lab.

Eukaryotic cell lines

Policy information about cell lines and Sex and Gender in Research

Cell line source(s)	The source of our cell lines: mouse LLC cell lines (ATCC, provided by Fei Li, Key Laboratory of Respiratory Disease of Zhejiang Province, Department of Respiratory and Critical Care Medicine, Second Affiliated Hospital of Zhejiang University School of Medicine), MC38 cell lines (ATCC, provided by Fei Li, Key Laboratory of Respiratory Disease of Zhejiang Province, Department of Respiratory and Critical Care Medicine, Second Affiliated Hospital of Zhejiang University School of Medicine), 4T1 cell lines (Cell Bank, Chinese Academy of Sciences,provided by Fei Li Key Laboratory of Respiratory Disease of Zhejiang Province, Department of Respiratory and Critical Care Medicine, Second Affiliated Hospital of Zhejiang University School of Medicine) and luciferace-transfected LLC (LLC-Luc) (provided by Dr. Chao Wan; Cancer Center, Union Hospital, Tongji Medical College, Huazhong University of Science and Technology).
Authentication	None of the cell lines used were authenticated.
Mycoplasma contamination	All cell lines tested negative for mycoplasma contamination every two week.
Commonly misidentified lines (See ICLAC register)	No commonly misidentified cell lines were used.

Animals and other research organisms

Policy information about studies involving animals; ARRIVE guidelines recommended for reporting animal research, and Sex and Gender in Research

Laboratory animals	Experimental animals used in the study comprised BALB/c, C57BL/6, Il5 Tg mice, and Eos-null mice, all aged between 6-8 weeks.
Wild animals	Animals were housed in a specific pathogen-free facility in the Zhejiang University animal center and the study did not involve any wild animals.
Reporting on sex	We selected female mice for experiments based on previous literature (), aiming to eliminate the influence of gender on experimental outcomes.
Field-collected samples	The study did not utilize samples collected from the field.
Ethics oversight	Animal experiments were conducted in strict accordance with the protocols approved by the Ethics Committee for Animal Studies at Zhejiang University (approval number: ZJU20220030).

Note that full information on the approval of the study protocol must also be provided in the manuscript.

Plants

Seed stocks

Report on the source of all seed stocks or other plant material used. If applicable, state the seed stock centre and catalogue number. If plant specimens were collected from the field, describe the collection location, date and sampling procedures.

Novel plant genotypes

Describe the methods by which all novel plant genotypes were produced. This includes those generated by transgenic approaches, gene editing, chemical/radiation-based mutagenesis and hybridization. For transgenic lines, describe the transformation method, the number of independent lines analyzed and the generation upon which experiments were performed. For gene-edited lines, describe the editor used, the endogenous sequence targeted for editing, the targeting guide RNA sequence (if applicable) and how the editor was applied.

Authentication

Describe any authentication procedures for each seed stock used or novel genotype generated. Describe any experiments used to assess the effect of a mutation and, where applicable, how potential secondary effects (e.g. second site T-DNA insertions, mosiacism, off-target gene editing) were examined.

Flow Cytometry

Plots

Confirm that:

☒ The axis labels state the marker and fluorochrome used (e.g. CD4-FITC).

☒ The axis scales are clearly visible. Include numbers along axes only for bottom left plot of group (a 'group' is an analysis of identical markers).

☒ All plots are contour plots with outliers or pseudocolor plots.

☒ A numerical value for number of cells or percentage (with statistics) is provided.

Methodology

Sample preparation

The pleural lavage or pleural effusion, obtained and centrifuged at 400g for 5 minutes at 4?, underwent red blood cell lysis using lysis buffer. Subsequently, PBS was added to create a single-cell suspension for use in subsequent flow cytometry experiments.

Instrument

CytoFlex analyzer (Beckman Coulter Life Science) .

Software

FlowJo 10.8.1 (BD Biosciences) or CytExpert 2.0 software (Beckman Coulter Life Science).

Cell population abundance

We mainly conducted microscopic evaluation to ensure the absence of contaminants or unwanted cell types.

Gating strategy

Using forward scatter (FSC) and side scatter (SSC) parameters to define individual cells, employing DAPI to exclude dead cells, and utilizing blank, negative, and single-color controls to delineate positive and negative staining cell populations.

☒ Tick this box to confirm that a figure exemplifying the gating strategy is provided in the Supplementary Information.
